# *Polygonatum cyrtonema* Hua polysaccharides alleviate muscle atrophy and fat lipolysis by regulating the gut microenvironment in chemotherapy-induced cachexia

**DOI:** 10.3389/fphar.2025.1503785

**Published:** 2025-03-10

**Authors:** Rongrong Zhou, Tingting Liu, You Qin, Jing Xie, Shuihan Zhang, Yi Xie, Jia Lao, Wei He, Hongliang Zeng, Xueyang Tang, Xuefei Tian, Yuhui Qin

**Affiliations:** ^1^ The Affiliated Hospital of Hunan Academy of Traditional Chinese Medicine, Changsha, China; ^2^ State Key Laboratory for Quality Ensurance and Sustainable Use of Dao-di Herbs, China Academy of Chinese Medical Sciences, Beijng, China; ^3^ Institute of Chinese Medicine Resources, Hunan Academy of Chinese Medicine, Changsha, China; ^4^ The ResGreen Group, Changsha, China; ^5^ School of Integrated Chinese and Western Medicine, Hunan University of Chinese Medicine, Changsha, China; ^6^ Hunan Province University Key Laboratory of Oncology of Traditional Chinese Medicine, Changsha, China

**Keywords:** *Polygonatum cyrtonema Hua polysaccharides*, chemotherapy-induced cachexia, muscle atrophy, gut microbiota, fecal metabolites, short-chain fatty acids

## Abstract

**Introduction:** *Polygonatum cyrtonema Hua* (PC) is an essential herbal medicine in China, known for improving muscle quality and enhancing physical function; its active ingredients are polysaccharides (PCPs). A previous study revealed the anti-atrophy effects of PCPs in cachectic mice. However, whether the effects of PCPs on anti-atrophy are associated with gut microenvironment remain elusive. This research endeavored to assess the medicinal efficacy of PCPs in alleviating muscle atrophy and fat lipolysis and explore the potential mechanisms.

**Methods:** A cancer cachexia model was induced by male C57BL/6 mice bearing Lewis lung tumor cells and chemotherapy. The pharmacodynamics of PCPs (32 and 64 mg/kg/day) was investigated through measurements of tumor-free body weight, gastrocnemius muscle weight, soleus muscle weight, epididymal fat weight, tissue histology analysis, and pro-inflammatory cytokines. Immunohistochemistry and Western blotting assays were further used to confirm the effects of PCPs. 16S rRNA sequencing, LC-MS and GC-MS-based metabolomics were used to analyze the gut microbiota composition and metabolite alterations. Additionally, the agonist of free fatty acid receptor 2 (FFAR2)—a crucial short-chain fatty acid (SCFA) signaling molecule—was used to investigate the role of gut microbiota metabolites, specifically SCFAs, in the treatment of cancer cachexia, with comparisons to PCPs.

**Results:** This study demonstrated that PCPs significantly mitigated body weight loss, restored muscle fiber atrophy and mitochondrial disorder, alleviated adipose tissue wasting, strengthened the intestinal barrier integrity, and decreased the intestinal inflammation in chemotherapy-induced cachexia. Furthermore, the reversal of specific bacterial taxa including *Klebsiella*, *Akkermansia*, *norank_f__Desulfovibrionaceae*, *Enterococcus*, *NK4A214_group*, *Eubacterium_fissicatena_group*, *Eubacterium_nodatum_group*, *Erysipelatoclostridium*, *Lactobacillus*, *Monoglobus*, *Ruminococcus*, *Odoribacter*, and *Enterorhabdus*, along with alterations in metabolites such as amino acids (AAs), eicosanoids, lactic acid and (SCFAs), contributed to the therapeutic effects of PCPs.

**Conclusion:** Our findings suggest that PCPs can be used as prebiotic drugs targeting the microbiome–metabolomics axis in cancer patients undergoing chemotherapy.

## 1 Introduction

Cancer cachexia is defined as a multifactorial metabolic syndrome related to a reduced functional state, decreased tolerance to chemotherapy, and poor survival rate (Fearon et al., 2011). It is estimated to affect up to 50%–80% of patients with advanced cancer and is directly responsible for over 30% of cancer-related deaths (Feng et al., 2021). Severe and ongoing weight loss, skeletal muscle atrophy, and adipose tissue wasting are recognized as characteristic features of cancer cachexia, which are directly attributable to systemic inflammation and metabolic disorders (Petruzzelli and Wagner, 2016). Notably, increased pro-inflammatory cytokines caused decreased mitochondrial function, both of which lead to accelerated catabolism and suppressed anabolism in cachexia-related muscle atrophy (Pin et al., 2019). One of the primary factors contributing to mitochondrial dysfunction is a reduction in mitochondrial respiratory capacity, which refers to the mitochondria’s capability to generate ATP to fulfill cellular energy requirements, such as those needed for protein synthesis (Powers et al., 2012). Therefore, if these inflammatory cytokines and/or genes can be regulated by medically functional materials, muscle atrophy development can be alleviated.

Various preclinical and human studies have shown that cancer and chemotherapy in cancer cachexia can lead to modifications in the composition of the gut microbiota and damage to the gut epithelial barrier (Ni et al., 2021; Panebianco et al., 2023; Ubachs et al., 2021). The dysregulation of the gut microenvironment could further influence the pathways of metabolic disorders and systemic inflammation, which exacerbate cancer cachexia. For instance, the dysbiosis of Gram-negative gut microbiota could lead to the elevation of lipopolysaccharide (LPS) and be associated with an acute inflammatory response (Tucureanu et al., 2018). In addition, gut microbiota has been used as a new therapeutic application in cancer cachexia, which could regulate muscle mass and function. The intervention of *Lactobacillus* probiotics, such as genera L. reuteri and L. gasseri, led to an increase in muscle mass and a decrease in the expression of key markers associated with muscular atrophy (atrogin-1, MuRF1, LC3, and cathepsin L) and the inflammatory cytokines (IL-4, MCP-1, G-CSF, and IL-6) (Bindels et al., 2012; Varian et al., 2018). Gut microbiota metabolites, such as short-chain fatty acids (SCFAs), facilitate communication between the gut and distant tissues. SCFAs play a role in improving insulin sensitivity, regulating inflammation (Canfora et al., 2015), and restoring muscle mass (Tang et al., 2022).

Polygonatum cyrtonema Hua (PC) is a widely distributed perennial plant widely distributed in East Asia and is used for tonifying qi, nourishing yin, resisting fatigue, and addressing the lack of strength (He et al., 2022). The main components of PC, Polygonatum cyrtonema Hua polysaccharides (PCPs), have received great attention due to their anti-inflammation (He et al., 2021), anti-cancer (Li et al., 2020), and anti-fatigue functions (Shen et al., 2021). According to the findings of Li et al. (2023), the use of PCPs promoted energy metabolism, ATP generation, and osteocalcin signaling in skeletal muscle, thereby alleviating fatigue in mice subjected to exhaustive swimming. In addition, Polygonatum polysaccharides had the potential to regulate the structure and composition of gut microbiota, which was related to its nutritional and medicinal value (Wang et al., 2018). Our previous study extracted and purified PCPs from Polygonatum cyrtonema Hua, and molecular weight, monosaccharide composition, and structure were identified. PCPs exhibited potent activity against atrophy in cancer cachexia mice through the pathways involved in the autophagy–lysosome system (ALS) and the ubiquitin–proteasome system (UPS) (Tang et al., 2023). Nonetheless, the underlying mechanism of PCPs with regard to the biochemical communication between the gut and muscles is required to be further investigated.

In this study, our objective was to assess the impact of PCPs on muscle atrophy in a cachexia mouse model induced by chemotherapy. The mechanism of PCPs for improving cancer cachexia symptoms has been investigated by 16S rRNA gene sequencing and UPLC-TOF-MS/MS combined with GC-MS. Furthermore, we used free fatty acid receptor FFAR2 (a crucial SCFA signaling molecule) agonist to investigate the role of gut microbiota metabolites, such as SCFAs, in the treatment of cancer cachexia, with comparisons to PCPs. The research findings suggest that PCP has the potential to aid in the management of cancer cachexia by modulating essential gut bacteria and their corresponding metabolites.

## 2 Material and methods

### 2.1 Materials and chemicals

*Polygonatum cyrtonema* Hua was provided by Hunan Xinhui Pharmaceutical Co., Ltd. (Hunan, China) and authenticated by associate Professor Hao Liu from the Hunan Academy of Chinese Medicine. The specimens of Polygonatum cyrtonema Hua (No. 430529-210703-1078LY) were collected at the Herbarium of the Institute of Chinese Medicine Resources, Hunan Academy of Chinese Medicine. The free fatty acid receptor FFA2 agonist (4-CMTB, HY-P1125) was purchased from MedChemExpress LLC (NJ, United States). Mouse IL-6 (ZC-37988), TNF-α (ZC-39024), NF-kB (ZC-39080), LPS (ZC-39007), and ELISA Kit were obtained from ZCIBIO Technology Co., Ltd (Wuhan, China). β-Actin (ab8226), ATP5A1 (ab14748), TOM20 (ab186735), COXIV (ab16056), and cytochrome C (ab133504) were provided by Abcam Co., Ltd (Cambridge, United Kingdom).

### 2.2 Separation and purification of P. Cyrtonema polysaccharide

According to the previous method described in the published literature (Tang et al., 2023), the crude PCP from *P. cyrtonema* was obtained by water extraction twice at 80°C, ethanol precipitation with a 75% ethanol solution, and D-101 macroporous resin purification at a mass ratio of 15 : 1. Then, the crude PCP was further purified using the Sevag solvent (consisting of 80% chloroform and 20% n-butanol in volume) at least five times until the protein emulsion layer disappeared. N-butanol solution was volatilized and lyophilized to obtain the PCP fraction. The purity of the PCP is 88.68% ± 2.50%, with the composition of arabinose (Ara), galactose (Gal), glucose (Glu), mannose (Man), xylose (Xyl), rhamnose (Rha), galacturonic acid (GalA), glucuronic acid (GluA), and mannuronic acid (ManA). The molecular weight of the PCP is 2.95 kDa.

### 2.3 Animals and drug administration

The Lewis lung cancer cell line (provided by the Cell Resource Center at the Shanghai Academy of Biology) was cultured, as described in the published paper (Tang et al., 2023). In brief, the cells were grown in DMEM with 10% FBS, 100 U/mL penicillin, and 100 U/mL streptomycin. In addition, the cells were cultured at 37°C in a 5% CO_2_ atmosphere. After the cells reached 80% density, 0.25% trypsin (EDTA+) was used to digest the cells. The cells were prepared for subsequent use at a density of 5 × 10^6^ cells/mL in serum-free DMEM solution.

All animal experiments were carried out based on the Guidelines for the Laboratory of Animal Center (Changsha, China, SYXK<Xiang>2022–0005) and the animal ethics protocols (Changsha, China, IACUC-XLH--2022–081) approved by the Hunan Academy of Chinese Medicine. Male C57BL/6 mice (6 weeks old; 20 ± 2 g) were provided by the Hunan Silaike Laboratory Animal Co., Ltd (Changsha, China) and kept under specific pathogen-free (SPF) conditions where they had free access to standard food and sterilized water under constant temperature (24°C ± 2°C) with 12 h light/dark cycles. After a week of accommodation, the mice were randomly divided into the Ctrl (control) group (n = 10) and the tumor-bearing mice group (n = 40). Forty mice were inoculated with 5 × 10^5^ LLC cells in the axilla of the left forelimb, while 10 mice in the Ctrl group were injected intraperitoneally with saline. The tumor-bearing mice group was further divided into four groups (10 mice per group), namely, GCL group, PL group (32 mg/kg/day PCP by gavage), PH group (64 mg/kg/day PCP by gavage), and FFAR2 group (0.2 mg/kg/day 4-CMTB by intraperitoneal injection). During the experiment, PCP was dissolved in distilled water, while the FFAR2 group was dissolved in 0.5% DMSO and administered to mice via oral gavage and intraperitoneal injection once a day, respectively. The Ctrl and GCL groups were administered an equivalent volume of distilled water. After tumor cell injection for 8 days, the GCL, PL, PH, and FFAR groups were treated with gemcitabine (60 mg/kg) and cisplatin (3 mg/kg) by intraperitoneal injection at 3-day intervals, totaling five occurrences. Body weight was measured on a weekly basis. In the fourth week, feces samples were collected in Eppendorf tubes kept on ice and then promptly stored at −80°C for sequence experiments. The mice were euthanized via an intraperitoneal injection of 60 mg/kg of sodium pentobarbital. Serum, spleen, thymus, ileum, muscle, and adipose tissue samples were collected for analysis. All experimental procedures were conducted in accordance with the Regulations of Experimental Animal Administration (Order No.2, approved by the State Council in 1988, Third revision in 2017) issued by the State Committee of Science and Technology of the People’s Republic of China and approved by the Institutional Animal Care and Use Committee (IACUC) of the Institute of Chinese Medicine, Hunan Academy of Chinese Medicine (Hunan, China).

### 2.4 Hematoxylin–eosin and immunohistochemical staining

The gastrocnemius (GA), epididymal adipose tissue, and jejunum were fixed in a 4% paraformaldehyde solution. They were then embedded in paraffin and frozen. The paraffin-embedded tissue specimens were sectioned and stained with hematoxylin–eosin (H&E). PANNORAMIC Digital Slide Scanners (3DHISTECH, Hungary) was used to observe and assess the muscle, adipose tissue cross-sectional area (CSA), and jejunum morphology. Immunohistochemical staining was conducted as follows: after deparaffinization, GA tissue sections were incubated at overnight 4°C with the corresponding primary antibodies: anti-rabbit atrogin-1 (1:500) (ServiceBio, GB11285) and anti-rabbit MuRF1 (1:1000) (ServiceBio, GB113448); epididymal adipose tissue sections were incubated overnight at 4°C with the corresponding primary antibody, UCP-1 (1:200) (ServiceBio, GB112174); and the jejunum sections were stained with the following primary antibodies: anti-rabbit claudin-1 (1:500) (ServiceBio, GB11032), anti-rabbit occludin (1:500) (ServiceBio, GB111401), and anti-rabbit ZO-1 (1:500) (ServiceBio, GB111402). Subsequently, the tissue sections incubated with horseradish peroxidase-conjugated secondary antibody, goat anti rabbit IgG (1:200) (ServiceBio, GB23404). Signal detection was accomplished using diaminobenzidine.

### 2.5 Western blotting

Western blotting was performed for the validation of protein expression levels in GA tissues. In brief, total protein was extracted using radioimmunoprecipitation assay lysis buffer and subsequently quantified using the Bicinchoninic Acid Protein Assay Kit. The protein was mixed with a 5× loading buffer and separated using 10.0% sodium dodecyl sulfate polyacrylamide gel electrophoresis. After the protein was transferred from the gel onto nitrocellulose membranes, TBST buffer containing 5.0% skimmed milk powder was applied to block the membranes for 1 h. The membrane was subjected to overnight incubation at 4°C with a primary antibody. Afterward, the membrane was incubated with the secondary antibody for 1 h at room temperature. The primary antibodies for ATP5A1, TOM20, COXⅣ, and cytochrome c were purchased from Abcam (UK). The relative protein levels were standardized against the level of β-actin, which served as the control. The results were obtained and analyzed using ImageJ software (National Institutes of Health).

### 2.6 Fecal DNA extraction and 16S rRNA amplicon sequencing

Total microbial genomic DNA was extracted using the E.Z.N.A. ^®^ soil DNA Kit (Omega Bio-Tek, Norcross, United States), according to the manufacturer’s instructions. The quality of the extracted genomic DNA was examined using 1% agarose gel electrophoresis, and DNA concentration and purity were determined using a NanoDrop^®^ ND-2000 (Thermo Scientific, United States) and stored at −80°C. The extracted DNA was used a as a template to amplify the 16S rRNA gene V3–V4 variable region using an ABI GeneAmp^®^ 9700 PCR Thermocycler (ABI, CA, United States), with the upstream primer 338F (5′-ACT​CCT​ACG​GGA​GGC​AGC​AG-3′) and downstream primer 806R (5′-GGACTACHVGGGTWTCTAAT-3′) carrying barcode sequence. The PCR system consisted of 4 μL of 5× TransStart FastPfu buffer, 2 μL of 2.5 mM dNTPs, 0.8 μL of the upstream primer (5 uM), 0.8 μL of the downstream primer (5 uM), 0.4 μL of TransStart FastPfu DNA polymerase, and 10 ng of template DNA, which was made up to a total volume of 20 μL. The amplification procedure was as follows: pre-denaturation at 95°C for 3 min, followed by 27 cycles ( denaturation at 95°C for 30 s, annealing at 55°C for 30 s, and extension at 72°C for 30 s), then a stable extension at 72°C for 10 min, and finally storage at 4°C (PCR instrument: ABI GeneAmp^®^ 9700 model). Three replicates were performed for each sample. PCR products from the same sample were pooled and recovered using a 2% agarose gel, purified using the AxyPrep DNA Gel Extraction Kit (Axygen Biosciences, United States), detected by 2% agarose gel electrophoresis, and quantified using a Quantus™ Fluorometer (Promega, United States) to measure the recovered products.

The data from each sample were normalized, and further analysis was based on the normalized data. Alpha diversity was assessed using the ACE index, which was calculated using mothur software (http://www.mothur.org/wiki/Calculators). Principal coordinate analysis (PCoA) based on Bray–Curtis distance was performed to test the similarity of microbial community structure between samples.

### 2.7 SCFA analysis of fecal samples

Gas chromatography–mass spectrometry (GC-MS) (Agilent, United States) was used to detect SCFAs in fecal samples. Chromatography separation was performed using an HP-FFAP capillary column (30 m × 0.25 mm × 0.25 μm, Agilent J&W Scientific, Folsom, CA, United States). High-purity helium (purity not less than 99.999%) was chosen as the carrier gas at a flow rate of 1.0 mL/min. The programmed temperature rise gradient was controlled as follows: the initial temperature of the GC column was held at 80°C, increased to 120°C at a rate of 20°C per min, and then increased to 160°C at a rate of 5°C per min, followed by a post-run at 220°C for 3 min.

Mass spectrometry was performed using an Agilent 5977B/7000D Mass Selective Detector combined with an inert electron impact (EI) ionization source. Ionization voltage was set at 70°eV (Agilent, United States). The temperature of the ion source, quadrupole, and transmission line was set at 230°C, 150°C, and 230°C, respectively, and electron energy was held at 70 eV.

The default parameters of MassHunter quantitative software (Agilent, United States, version number: v10.0.707.0) were used to automatically identify and integrate each ion fragment of the target short-chain fatty acid and assist manual inspection. The actual content of short-chain fatty acids in each sample was calculated according to the standard curve.

### 2.8 Metabolomic profiling of fecal samples

Non-targeted metabolomics in fecal samples were analyzed using an ultra-high performance liquid chromatography-tandem Fourier transform mass spectrometer (UHPLC-Q Exactive HF-X) (Thermo Scientific, United States). Chromatography separation was performed using an HSS T3 Column (100 mm × 2.1 mm i. d., 1.8 µm, Agilent J&W Scientific, Folsom, United States) at a temperature of 40°C. Using 95% water plus 5% acetonitrile (containing 0.1% formic acid) as the aqueous phase (mobile phase B) and 47.5% acetonitrile plus 47.5% isopropanol and 5% water (containing 0.1% formic acid) as the organic phase (mobile phase A), and the flow rate was 0.40 mL/min. The gradient elution procedure of positive ion mode was set as follows: 0–3 min, 0%–20% B; 3–4.5 min, 20%–35% B; 4.5–5 min, 35%–100% B; 5–6.3 min, maintained at 100% B; 6.3–6.4 min, decreased from 100% to 0% B; and 6.4–8 min, maintained at 0% B. In addition, a separation gradient in negative ion mode was performed as follows: 0–1.5 min, 0%–5% B; 1.5–2 min, 5%–10% B; 2–4.5 min, 10%–30% B; 4.5–5 min, 30%–100% B; 5–6.3 min, maintained at 100% B; 6.3–6.4 min, decreased from 100% to 0% B; and 6.4–8 min, maintained at 0% B.

The mass spectrometry signal acquisition of fecal samples was performed in positive and negative ion scan modes with the following parameter settings: the mass scan range was 70–1050 m/z, the sheath gas flow rate was 50 psi, the auxiliary gas flow rate was 13 psi, the auxiliary gas heating temperature was 425°C, the positive mode ion spray voltage was set to 3500 V, the negative mode ion spray voltage was set to −3500 V, the ion transfer tube temperature was 325°C, and the normalized collision energy was 20–40–60 V cyclic collision energy. The primary mass spectrometry resolution was 60,000, and the secondary mass spectrometry resolution was 7,500. Data were collected in DDA mode.

Databases including the HMDB (http://www.hmdb.ca/), METLIN (https://metlin.scripps.edu/), and Majorbio were searched to match the metabolites detected by the UHPLC-Q Exactive HF-X system. The data matrix obtained by searching the databases was then uploaded to the Majorbio cloud platform (https://cloud.majorbio.com) for data analysis, including principal component analysis (PCA) and partial least squares discriminant analysis (PLS-DA). Significantly different metabolites were selected based on the variable importance in projection (VIP) value obtained from the established PLS-DA model and Student’s t-test P-value, with criteria set at VIP>1 and P < 0.05. The pathways associated with the differential metabolites were obtained through metabolic pathway annotation from the KEGG database (https://www.kegg.jp/kegg/pathway.html). Potential biomarkers were screened based on VIP value > 1.0, P < 0.05, and fold change >1.2 or <5/6. The obtained differential metabolites were used to perform pathway analysis using MetoboAnalyst 5.0.

### 2.9 Statistical analysis

The data were presented as the mean ± SEM. SPSS 22.0 software (IBM Corp, United States) was used for data analysis. Student’s t-test was applied to analyze the differences between the two groups. A one-way ANOVA test and Duncan’s new multiple-range test were chosen to measure the statistical significance between groups. Spearman’s rank correlation tests were applied to assess the correlations among the microbiota, fecal metabolites, SCFAs, and cancer cachectic traits.

## 3 Results

### 3.1 PCPs prevented chemotherapy-induced body weight loss in the cachexia mouse model

**FIGURE 1:**
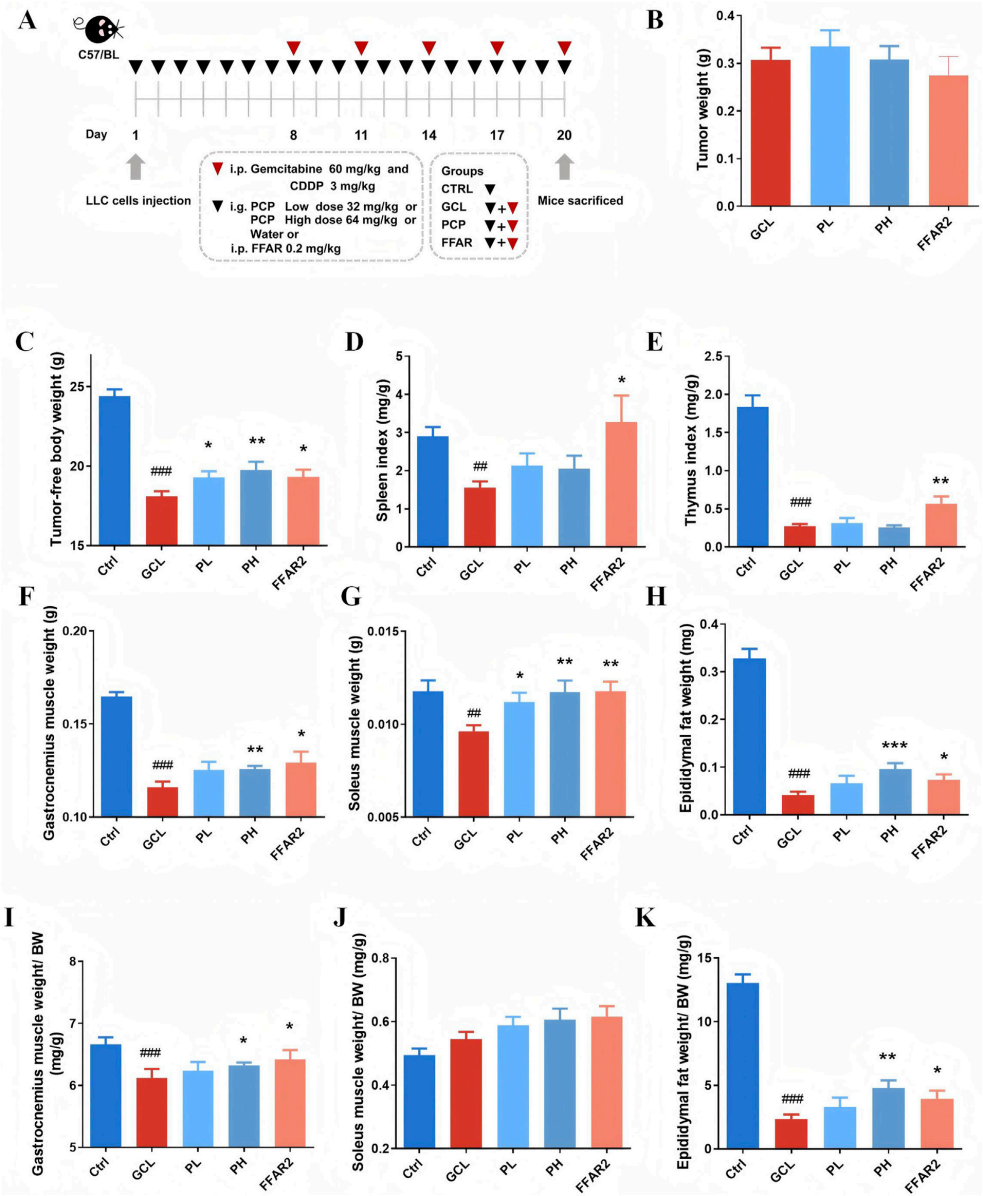
PCPs prevented chemotherapy-induced body weight loss in the cachexia mouse model (n = 10). **(A)** Animal experimental design. **(B)** Tumor weight. **(C)** Tumor-free body weight. **(D)** Spleen index. **(E)** Thymus index. **(F)** Gastrocnemius muscle weight. **(G)** Soleus muscle weight. **(H)** Epididymal fat weight. **(I)** Relative ratio of gastrocnemius muscle weight to body weight. **(J)** Relative ratio of soleus muscle weight to body weight. **(K)** Relative ratio of epididymal fat weight to body weight. The data are presented as the mean ± SEM. #*P* < 0.05, ##*P* < 0.01, ###*P* < 0.001 (vs*.* Ctrl group); *P < 0.05, **P < 0.01, ***P < 0.001 (vs*.* GCL group).

It is known that cancer cachexia is characterized by a severe reduction in weight, skeletal muscle, and adipose. Therefore, we monitored these three phenotypic indicators to observe the effect of PCPs on cachexia mice initially. As shown in Figure 1C–K, significant decreases in the weight of tumor-free body (*P* < 0.001), gastrocnemius muscle (*P* < 0.001), soleus muscle (*P* < 0.01), epididymal fat (*P* < 0.001), the relative gastrocnemius muscle weight ratio (*P* < 0.001), and the relative ratio of epididymal fat weight to body weight (*P* < 0.001) were observed in the GCL group compared with the Ctrl group. The relative soleus muscle weight ratio showed no significant variation between the Ctrl and GCL groups. These results demonstrated a typical cachectic pathological status. In addition, we also observed a significant decrease in the indexes of spleen (*P* < 0.01) and thymus (*P* < 0.001) the GCL group, which was consistent with previous studies (Tang et al., 2023), highlighting the immunosuppressive effect of chemotherapy drugs. In the treatment groups, the low-dose PCP supplementation (32 mg/kg/day, gavage) dose reversed weight loss in the body (*P* < 0.05) and soleus muscle (*P* < 0.05). The high-dose PCP (64 mg/kg/day, gavage) showed a more significant effects, including an increase in the body weight (*P* < 0.01), gastrocnemius muscle weight (*P* < 0.01), soleus muscle weight (*P* < 0.01), epididymal fat (*P* < 0.001), the relative ratio of gastrocnemius muscle weight to body weight (*P* < 0.05), and the relative ratio of epididymal fat weight to body weight (*P* < 0.01). The reference agent FFAR2 exerted protective effects on tumor-free body weight (*P* < 0.05), gastrocnemius muscle (*P* < 0.05), soleus muscle (*P* < 0.01), epididymal fat (*P* < 0.05), the relative ratio of gastrocnemius muscle weight to body weight (*P* < 0.05), the relative ratio of epididymal fat weight to body weight (*P* < 0.05), spleen index (*P* < 0.05), and thymus index (*P* < 0.01) in cancer cachectic mice.

#### 3.2 PCP ameliorated muscle fiber atrophy and adipose tissue wasting in the cachexia mouse model

**FIGURE 2:**
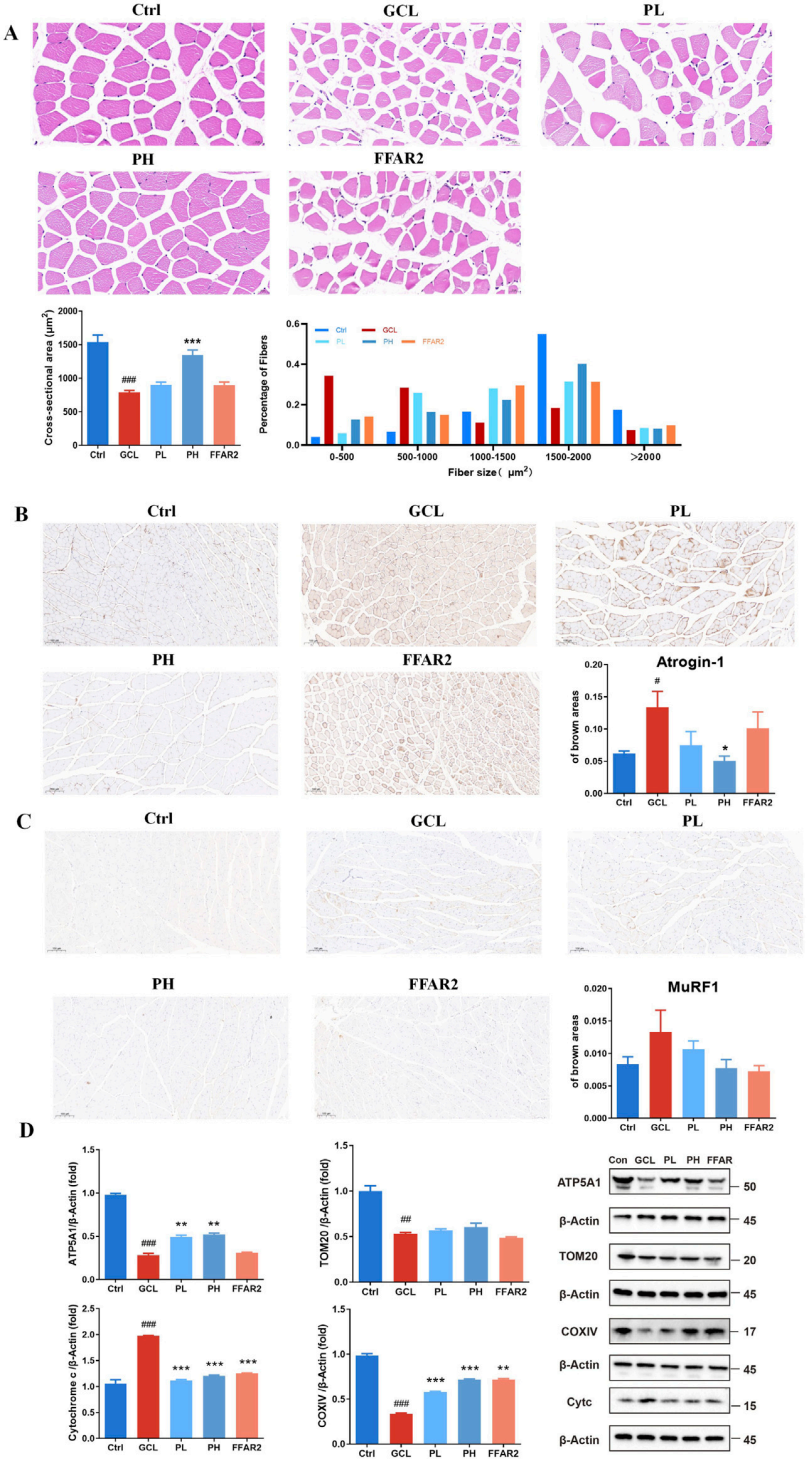
PCP ameliorated muscle fiber atrophy in the cachexia mouse model. **(A)** HE staining of muscle section (40×; scale bar = 20 μm); CSA of gastrocnemius muscle fibers; the proportion of CSA of gastrocnemius muscle fibers. **(B)** Immunohistochemistry staining of muscle section with atrogin-1 (10×; scale bar = 100 μm). **(C)** Immunohistochemistry staining of muscle section with MuRF1 (10×; scale bar = 100 μm) (n = 6). **(D)** Protein expression of ATP5A1, TOM20, COXⅣ, and cytochrome c in muscle tissue. (n = 3). The data are presented as the mean ± SEM. *#P* < 0.05, *##P* < 0.01, ###*P* < 0.001 (vs*.* Ctrl group); *P < 0.05, **P < 0.01, ***P < 0.001 (vs*.* GCL group).

The results of H&E and immunohistochemical tests provided a more visual depiction of variations in gastrocnemius tissue fibers and adipose tissue. As demonstrated in Figure 2A, the CSA in the GCL group of cachectic muscle fiber showed a significant decrease (*P* < 0.001). The mean CSA of GA fiber was 790 μm^2^ in the GCL group, which was approximately 51.4% of the normal CSA, indicating muscle atrophy. The CSA of muscle fiber was notably larger after high-dose PCP supplementation than that in the GCL group (*P* < 0.001). The average CSA of the PH group reverted to 1346 μm^2^, which was 1.70 times that of the GCL group and 0.88 times that of the Ctrl group, respectively. Moreover, the distribution of gastrocnemius muscle fiber sizes in the GCL group demonstrated a significant shift toward smaller fiber sizes than that in the Ctrl group. Following PH treatment, the proportion of larger muscle fibers increased, while the proportion of smaller fibers decreased, resulting in a distribution pattern more comparable to that of the Ctrl group. It implicated a protective effect of PCP treatment on muscle atrophy in a chemotherapy-related cachexia mouse model. Figures 2B, C show an increased expression of atrogin-1 (*P* < 0.05) and a tendency of increased MuRF1 expression in the skeletal muscle of cancer cachectic mice. After a high-dose PH treatment, both the levels of atrogin-1 (*P* < 0.05) and MuRF1 were reduced. Mitochondrial dysfunction has been documented in various cancer cachectic models. Mutations in either the nuclear or mitochondrial genome could induce the dysfunction of the mitochondrial electron transport chain (ETC) and further induce protein catabolism. Hence, the Western blot analysis of nuclear-encoded ETC components ATP5A1, COXⅣ, TOM20, and cytochrome c has been demonstrated (Figure 2D). The GCL treatment resulted in a decrease in the relative protein of ATP5A1 (*P* < 0.001), COXⅣ (*P* < 0.001), and TOM20 (*P* < 0.01) and an increase in the relative protein of cytochrome c (*P* < 0.001) compared to the Ctrl group. In contrast to the GCL group, both the PL and PH groups reversed the mitochondrial dysfunction in cancer cachectic muscle and showed a dose-dependent effect. The PH group markedly elevated the relative protein levels of ATP5A1 (*P* < 0.01) and COXⅣ (*P* < 0.001), showed an increasing trend in TOM20 (*P* > 0.05), resulted in a significant reduction in cytochrome c (*P* < 0.001).

**FIGURE 3:**
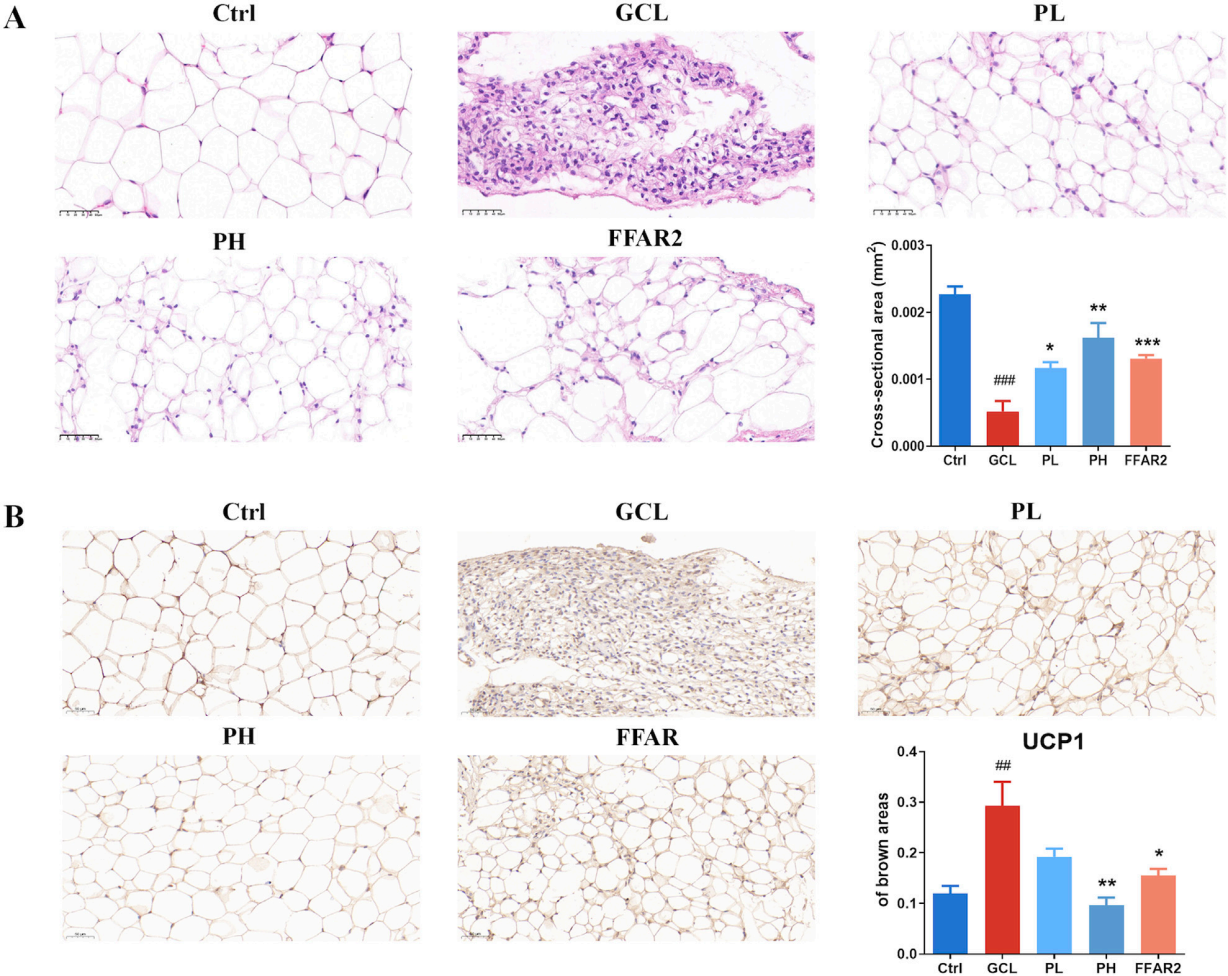
PCP mitigated adipose tissue wasting in the cachexia mouse model (n = 6). **(A)** HE staining of adipose tissue section (20×; scale bar = 50 μm). **(B)** Immunohistochemistry staining of adipose tissue section with UCP1 (20×; scale bar = 50 μm). The data are presented as the mean ± SEM. #*P* < 0.05, ##*P* < 0.01, ###*P* < 0.001 (vs*.* Ctrl group); *P < 0.05, **P < 0.01, ***P < 0.001 (vs*.* GCL group).

In addition, for the epididymal fat tissue, representative HE staining is shown in Figure 3A. There were noticeable morphological distinctions among the different groups, with the adipose tissue in the GCL group showing severe shrinkage. By quantifying the cross-sectional area, it was found that the adipose tissue in tumor-bearing mice was significantly reduced in size (*P* < 0.001), while PL (*P* < 0.05), PH (*P* < 0.01), gavage, and FFAR2 (*P* < 0.001) predominantly prevented fat loss. Uncoupling protein-1 (UCP1) IHC staining analysis of gonadal WAT showed an obvious phenotypic switch from white to brown adipose, and the percentage of the brown area increased from 0.11 to 0.29 in cachectic mice (*P* < 0.01, Figure 3B), which is consistent with the previous finding of cancer cachexia in mice (Petruzzelli et al., 2014). PCP supplementary (*P* < 0.01) ameliorated the evident WAT browning indicated by the decrease in brown areas compared to the GCL group. These results indicated that the efficacy of PCPs on chemotherapy-associated body weight loss, muscle fiber atrophy, and adipose tissue wasting in a cachexia mouse model exhibited a dose-dependent trend, similar to the effect of FFAR2.

##### 3.3 PCPs alleviated chemotherapy-induced gut microenvironment in the cachexia mouse model

###### 3.3.1 PCPs reversed the ileum injury and inflammation in the cachexia mouse model

**FIGURE 4:**
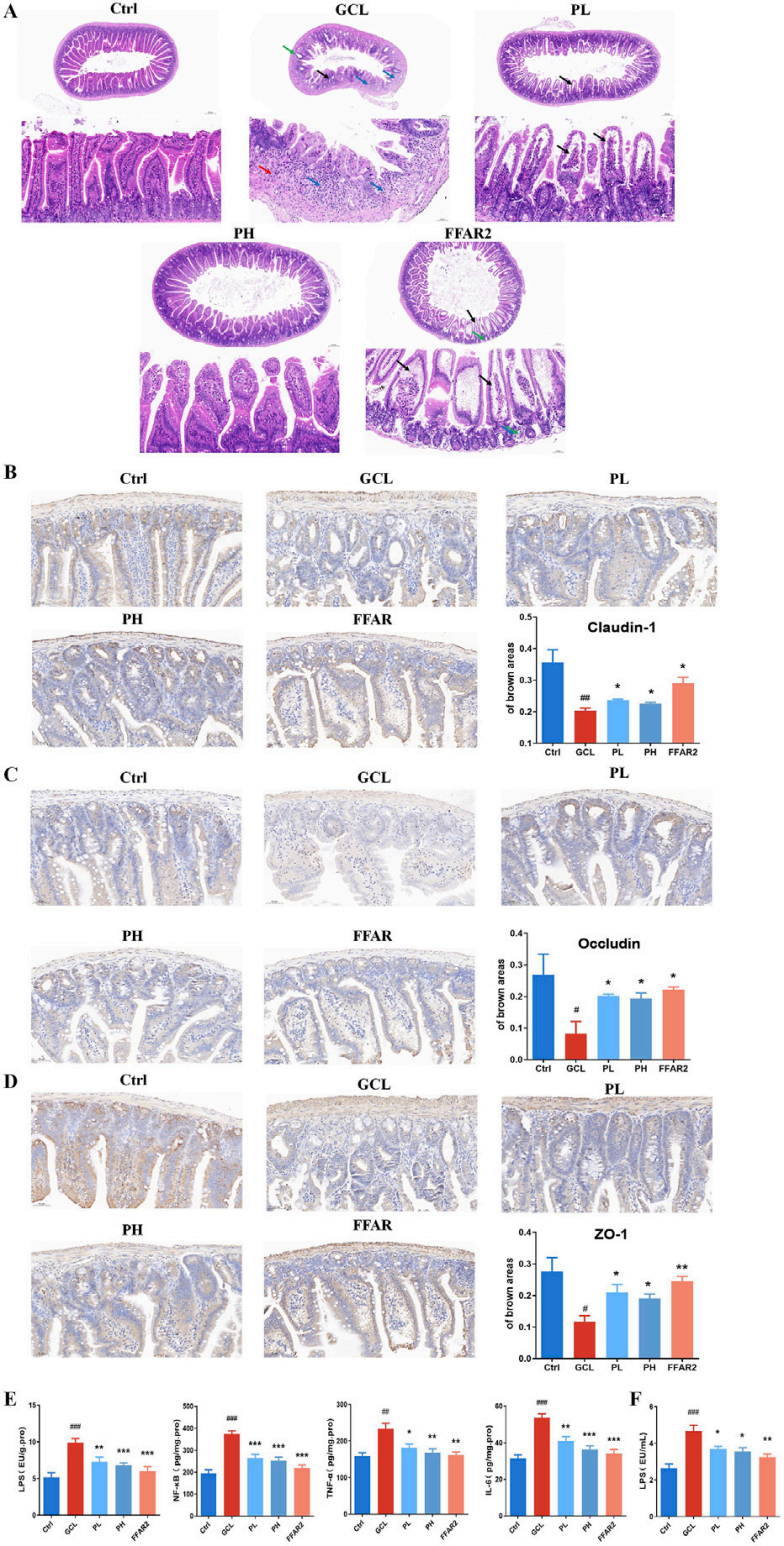
PCP reversed the ileum injury and inflammation in the cachexia mouse model. **(A)** HE staining of the ileum section (5× and scale bar = 200 μm; 20× and scale bar = 50 μm). **(B)** Immunohistochemistry staining of the ileum section with claudin-1 (20×; scale bar = 50 μm). **(C)** Immunohistochemistry staining of the ileum section with occludin (20×; scale bar = 50 μm). **(D)** Immunohistochemistry staining of the ileum section with ZO-1 (20×; scale bar = 50 μm). (n = 6). **(E)** Pro-inflammatory cytokines (LPS, IL-6, TNF-α, and NF-κB) of ileum tissue (n = 8). **(F)** LPS of serum (n = 10). The data are presented as the mean ± SEM. #*P* < 0.05, ##*P* < 0.01, ###*P* < 0.001 (vs*.* Ctrl group); *P < 0.05, **P < 0.01, ***P < 0.001 (vs*.* GCL group).

To confirm the protective effect of PCPs on the mucosa of the ileum, HE staining was performed (Figure 4A). Compared to the Ctrl group, the intestinal barrier was evidently disrupted in the GCL group, as shown by a reduction in the thickness of the mucus layer, destruction of the intestinal gland structure, and an increased number of infiltrating lymphocytes and granulocytes. In contrast, in the PCP group, the thickness of the mucus layer increased, and the intestinal glands were more organized and tightly arranged. The levels of tight junction proteins, including integral transmembrane proteins such as claudin-1 (*P* < 0.01) and occludin (*P* < 0.05) and peripheral membrane adapter proteins such as zonula occludens-1 (ZO-1, *P* < 0.05), were decreased in mice treated with GCL, as indicated by immunohistochemical staining (Figures 4B–D). Compared to the GCL group, PCP markedly increased claudin-1 (*P* < 0.05), occludin (*P* < 0.05), and ZO-1 (*P* < 0.05) levels, while the FFAR2 group exhibited a significant increase in the protein level of claudin-1 (*P* < 0.05), occludin (*P* < 0.05), and ZO-1 (*P* < 0.01).

Additionally, pro-inflammatory factors (including NF-κB, TNF-α, and IL-6) in cecal tissue, along with endotoxin (LPS) levels in both the serum and cecal tissues, were analyzed to assess localized inflammation within the intestinal barrier (Figure 4E) and systemic inflammation (Figure 4F). In cachectic mice of the GCL group, significant elevation of all four factors were monitored versus the Ctrl group, with a fold change of 1.9 (LPS in cecal, *P* < 0.001), 1.9 (LPS in serum, *P* < 0.001), 1.9 (NF-κB, *P* < 0.001), 1.4 (TNF-α, *P* < 0.01), and 1.7 (IL-6, *P* < 0.001). This inflammatory environment, induced by cancer cachexia, plays a major role in cachexia pathophysiology (Fearon et al., 2012). After PCP supplementation, the concentrations of four factors in the cecal tissue and serum significantly decreased, returning to normal levels, with a dose-dependent effect.

##### 3.3.2 PCPs reshaped fecal gut microbiota dysbiosis in the cachexia mouse model

**FIGURE 5:**
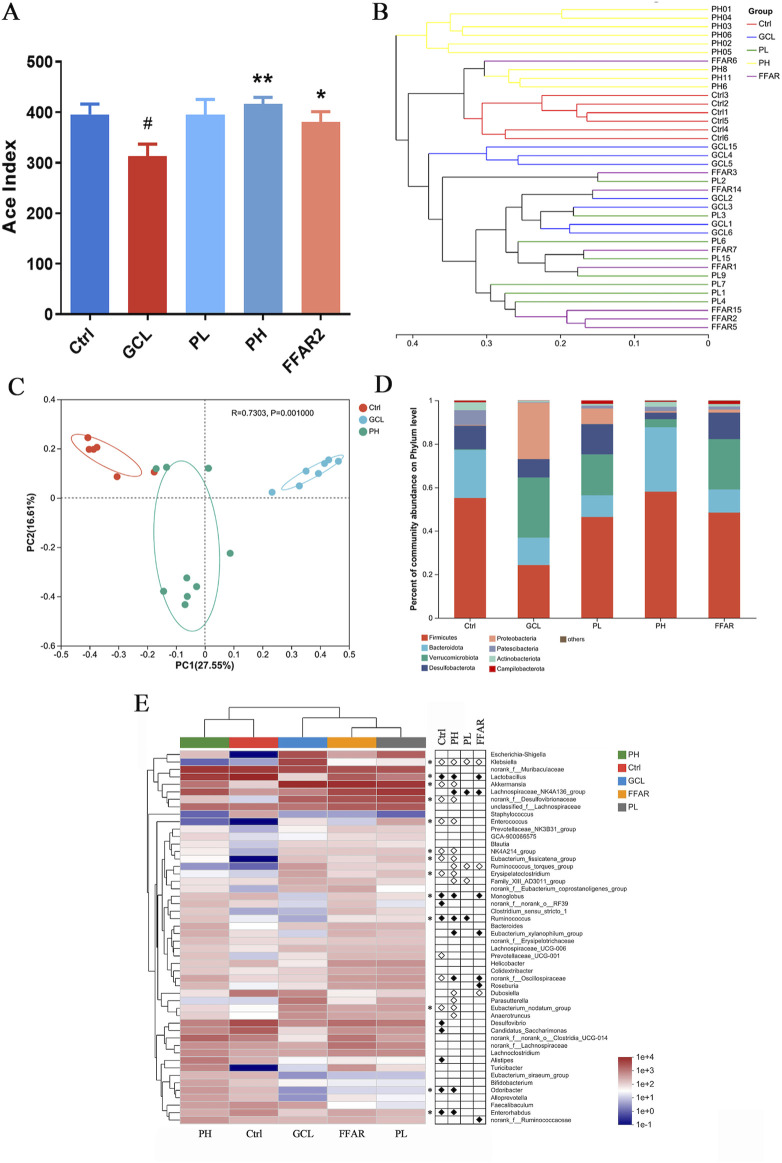
PCP reshaped the fecal gut microbiota dysbiosis in the cachexia mouse model. (n = 6–9). **(A)** ACE index. The data are presented as the mean ± SEM. *#P* < 0.05, ##*P* < 0.01, ###*P* < 0.001 (vs*.* Ctrl group); *P < 0.05, **P < 0.01, ***P < 0.001 (vs*.* GCL group. **(B)** Hierarchical clustering. **(C)** Bray–Curtis PCoA. **(D)** Taxonomic distribution of bacterial communities at the phylum level. **(E)** Heatmap of species abundance at the genus level (top 50 bacteria). White circles indicate genera that are less abundant in the Ctrl, PL, PH, and FFAR groups compared to the GCL group, *P* < 0.05. Conversely, black diamonds signify genera that are more abundant in the Ctrl, PL, PH, and FFAR groups relative to the GCL group, *P* < 0.05. Asterisks denote genera whose abundance in the Ctrl mice was reduced by GCL and subsequently modulated by PH.

Based on 16S rRNA gene technology, the importance of PCPs in maintaining gut microbiota was demonstrated. Alpha diversity of gut microbiota was analyzed using the ACE index, as shown in Figure 5A. The GCL treatment resulted in a significant reduction (*P* < 0.05) in the ACE index compared to the control group. Following high-dose PCP treatment, the diversity and evenness of the microbial community showed a significant reversal (*P* < 0.01) compared to the GCL group. The β-diversity of microbial composition (Figures 5B, C) among different groups was calculated using Bray–Curtis distance and imaged using PCoA. Clear distinctions were discernible among the Ctrl, GCL, and PH groups.

Next, we focused on taxonomic composition at different levels. At the phylum level (Figure 5D), the GCL treatment significantly decreased the abundance of *Firmicutes* (*P* < 0.001), *Patescibacteria* (*P* < 0.05), and *Actinobacteria* (*P* < 0.01) and significantly increased the abundance of *Verrucomicrobiota* (*P* < 0.05) and *Proteobacteria* (*P* < 0.01) compared to the Ctrl group. In contrast to the GCL group, the PH group has a significant decrease in *Verrucomicrobiota* (*P* < 0.05), *Proteobacteria* (*P* < 0.01), and *Desulfobacterota* (*P* < 0.05) and a significant increase in *Firmicutes* (*P* < 0.001)*, Bacteroidota* (*P* < 0.05), and *Actinobacteria* (*P* < 0.05)*.* Notably, treatment with PH reversed *Verrucomicrobiota, Proteobacteria*, *Firmicutes,* and *Actinobacteria*, which were disturbed by GCL. At the genus level, a heatmap of the top 50 genera abundance clusters across all experimental groups was generated (Figure 5E). The comparative analysis revealed significant alterations in the relative abundance of 19 genera (GCL vs. Ctrl group, *P* < 0.05) and 21 genera (GCL vs. PH group, *P* < 0.05). Notably, compared to the Ctrl group, the GCL group exhibited a higher abundance of *Klebsiella*, *Akkermansia*, *norank_f__Desulfovibrionaceae*, *Enterococcus*, *NK4A214_group*, *Eubacterium_fissicatena_group*, *Erysipelatoclostridium*, *Prevotellaceae_UCG-001*, *unclassified_f__Oscillospiraceae*, and *Eubacterium_nodatum_group*, while the abundance of *Lactobacillus*, *Monoglobus*, *norank_f__norank_o__RF39*, *Ruminococcus*, *Desulfovibrio*, *Candidatus_Saccharimonas*, *Alistipes*, *Odoribacter*, and *Enterorhabdus* decreased*.* Moreover, compared to the GCL group, the genera of *Klebsiella*, *Akkermansia*, *norank_f__Desulfovibrionaceae*, *Enterococcus*, *NK4A214_group*, *Eubacterium_fissicatena_group*, *Ruminococcus_torques_group*, *Erysipelatoclostridium*, *Family_XIII_AD3011_group*, *Dubosiella*, *Parasutterella*, *Eubacterium_nodatum_group*, and *Anaerotruncus* were reduced, while the genera of *Lactobacillus, Lachnospiraceae_NK4A136_group*, *Monoglobus*, *Ruminococcus*, *Eubacterium_xylanophilum_group*, *norank_f__Oscillospiraceae*, *Odoribacter*, and *Enterorhabdus* increased after PH treatment. PH treatment markedly reversed the increase in *Klebsiella*, *Akkermansia*, *norank_f__Desulfovibrionaceae*, *Enterococcus*, *NK4A214_group*, *Eubacterium_fissicatena_group*, *Eubacterium_nodatum_group*, *Erysipelatoclostridium* and the decrease in *Lactobacillus*, *Monoglobus*, *Ruminococcus*, *Odoribacter*, and *Enterorhabdus* caused by GCL treatment (indicated by asterisks in Figure 5E).

##### 3.3.3 PCPs modulated the fecal metabolomics and SCFA disorders in the cachexia mouse model

**FIGURE 6:**
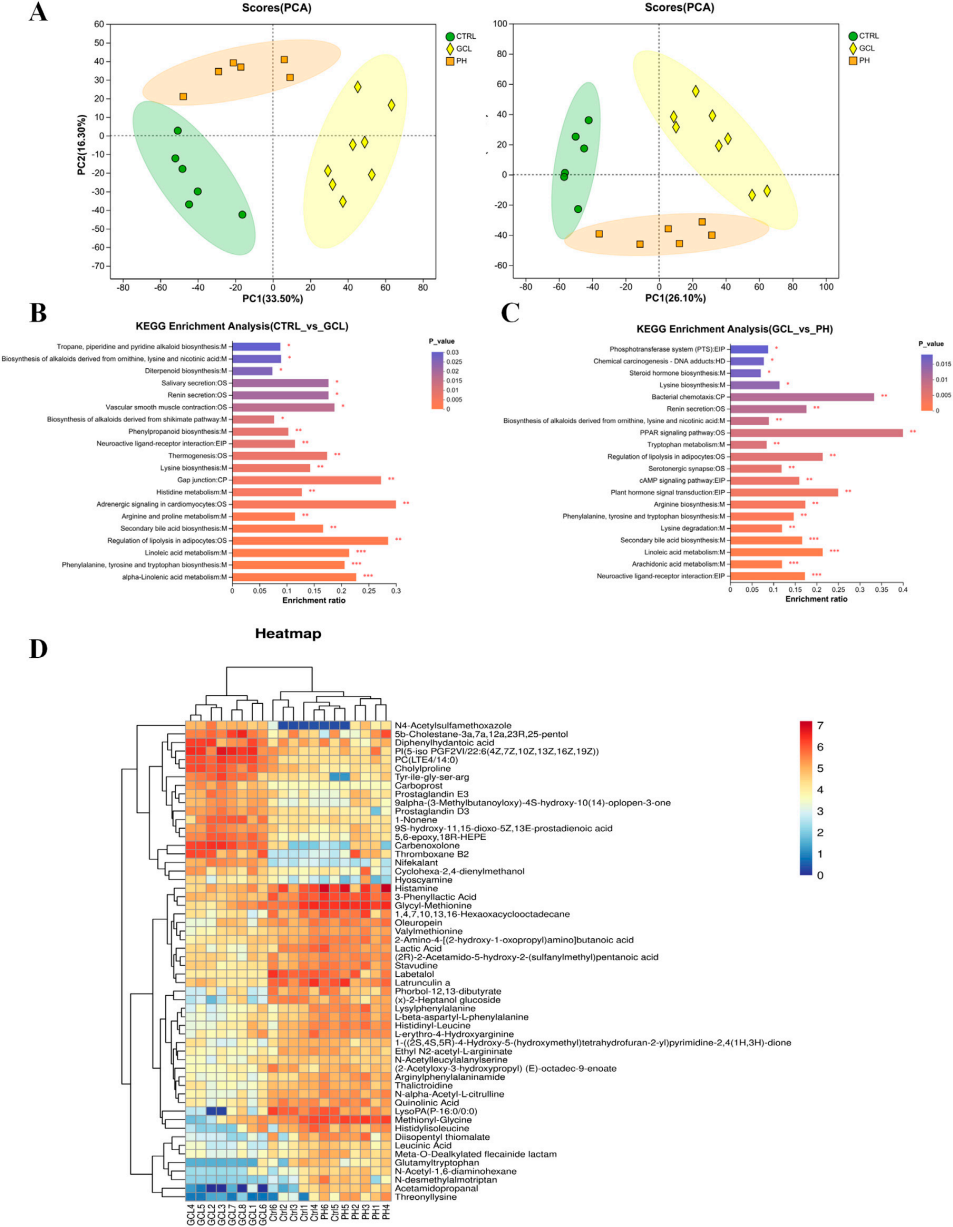
PCP attenuated the fecal metabolomic dysregulation in the cachexia mouse model (n = 6–8). **(A)** PCA among Ctrl, GCL, and PH. **(B)** KEGG annotation analysis (Ctrl vs. GCL). **(C)** KEGG annotation analysis (GCL vs. PH). **(D)** Heatmap analysis of 56 intersecting metabolites between Ctrl vs. GCL and PH vs. GCL.

To further explore the corresponding fecal metabolites induced by alterations in gut microbiota, we conducted a comparative analysis and filtration of differentially expressed metabolites. Untargeted metabolomics in positive and negative ion modes has recognized a total of 2,827 metabolites. PCA (Figure 6A) revealed global metabolic distinctions among the Ctrl, GCL, and PH groups. To further analyze the metabolic pathways of high-dose PCP for regulating chemotherapy-induced cancer cachexia, the KEGG enrichment analysis was further applied using MetaboAnalyst (www.metaboanalyst.ca), and Figures 6B, C show the top 20 metabolic pathways. Therefore, compared to the Ctrl with GCL groups, alpha-linolenic acid metabolism, phenylalanine, tyrosine, and tryptophan biosynthesis, linoleic acid metabolism, the regulation of lipolysis in adipocytes, secondary bile acid biosynthesis, arginine and proline metabolism, adrenergic signaling in cardiomyocytes, histidine metabolism, gap junction, lysine biosynthesis, thermogenesis, neuroactive ligand–receptor interactions, and phenylpropanoid biosynthesis were significantly enriched (*P* < 0.01). Neuroactive ligand–receptor interaction, arachidonic acid metabolism, linoleic acid metabolism, secondary bile acid biosynthesis, lysine degradation, phenylalanine, tyrosine and tryptophan biosynthesis, arginine biosynthesis, plant hormone signal transduction, the cAMP signaling pathway, serotonergic synapse, the regulation of lipolysis in adipocytes, tryptophan metabolism, the PPAR signaling pathway, the biosynthesis of alkaloids derived from ornithine, lysine, and nicotinic acid, renin secretion, and bacterial chemotaxis emerged as pivotal signaling pathways in the PH treatment in contrast to the GCL group (*P* < 0.01). Through cross-comparisons of PH vs. GCL and GCL vs. CTRL group, both the GCL and PH groups had great effects on phenylalanine, tyrosine and tryptophan biosynthesis, linoleic acid metabolism, regulation of lipolysis in adipocytes, secondary bile acid biosynthesis, and neuroactive ligand–receptor interactions.

To further elucidate the biomarkers across the groups, PLS-DA was employed to distinguish between them. In addition, 243 (Ctrl vs. GCL) and 116 (GCL vs. PH) endogenous metabolites with VIP >1, fold change >1.2 or <5/6, and *P* < 0.05 were selected as important features and are listed in Supplementary Table S1. Among them, 56 differentially regulated metabolites exhibited overlap between Ctrl vs. GCL and PH vs. GCL (Figure 6D). Notably, the administration of PH reversed all the metabolic biomarkers with 37 upregulated and 19 downregulated, which were disrupted by GCL. The upregulated metabolites displayed overlaps in amino acids, peptides, and analogs (14/37), while the downregulated metabolites exhibited overlaps in eicosanoids. This suggests that PH treatment alters cancer cachexia-associated metabolism in GCL mice.

**FIGURE 7:**
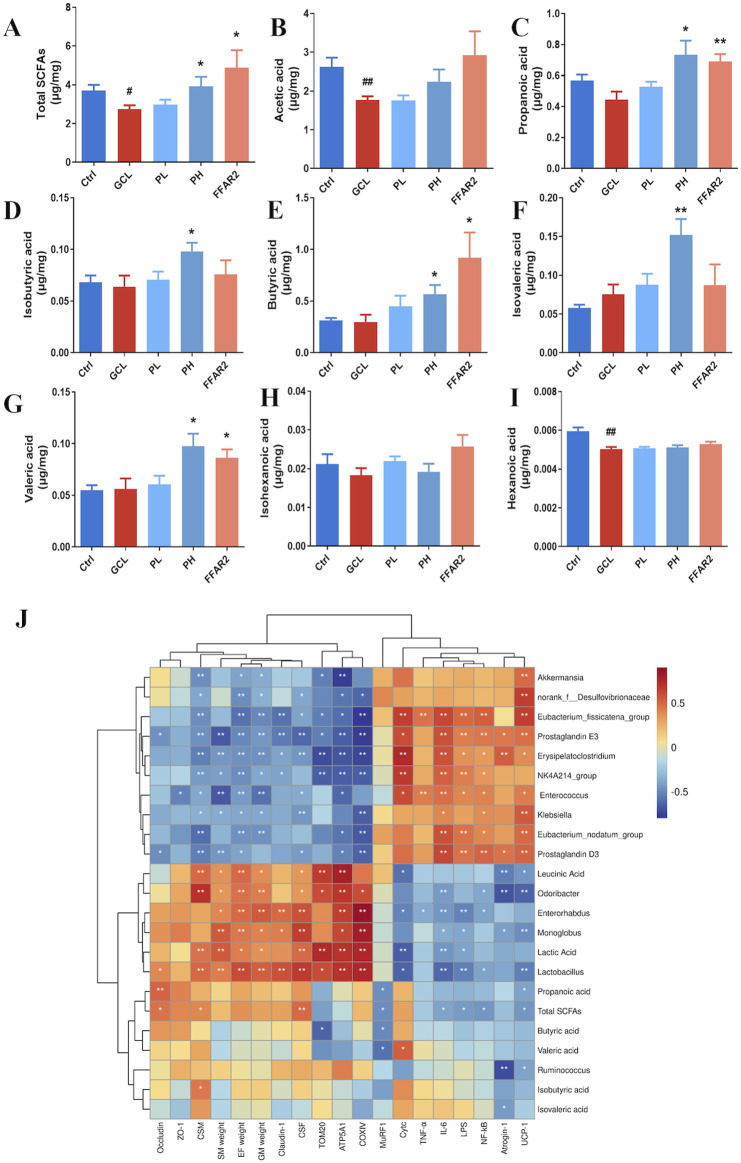
PCP modulated the SCFA metabolism disorders in the cachexia mouse model. (n = 8). **(A)** Total SCFAs. **(B)** Acetic acid. **(C)** Propanoic acid. **(D)** Isobutyric acid. **(E)** Butyric acid. **(F)** Isovaleric acid. **(G)** Valeric acid. **(H)** Isohexanoic acid. **(I)** Hexanoic acid. **(J)** Heatmap analysis of Spearman correlation coefficients for the relationship between bacterial genera, metabolites, and phenotype. Data are presented as the mean ± SEM. *#P* < 0.05, ##*P* < 0.01, ###*P* < 0.001 (vs*.* Ctrl group); *P < 0.05, **P < 0.01, ***P < 0.001 (vs*.* GCL group).

We further examine the cecal SCFA contents among the five groups (Figures 7A–I). Compared to the Ctrl groups, the total SCFAs (*P* < 0.05), acetic acid (*P* < 0.01), and hexanoic acid (*P* < 0.01) showed significantly lower levels in GCL mice treated with chemotherapy. Compared to the GCL group, the PH group increased the total SCFAs (*P* < 0.05), propanoic acid (*P* < 0.05), isobutyric acid (*P* < 0.05), butyric acid (*P* < 0.05), isovaleric acid (*P* < 0.01), and valeric acid (*P* < 0.05). In contrast to the GCL group, the FFAR2 group showed relatively higher total SCFA levels (*P* < 0.05), propanoic acid (*P* < 0.01), butyric acid (*P* < 0.05), and valeric acid (*P* < 0.05). The results validated the function of the free fatty acid receptor 2 agonist, which increased the activity of SCFAs.

### 4 Discussion

Tumor promotes tissue wasting, mobilizes metabolites, and ultimately provides support for its growth that induces systemic metabolic dysregulation (Porporato, 2016). Chemotherapy represents the primary anticancer strategy for treating cancer. However, the non-specific cytotoxicity of chemotherapy often leads to a series of side effects in systemic toxicities and gastrointestinal issues (Chen et al., 2007). These side effects result in poor appetite and diarrhea in patients, which deteriorate the cachectic phenotype, with weight loss being the most prominent feature (Amrute-Nayak et al., 2021). Therefore, in the current study, a cachexia mouse model was established by injecting mice with Lewis lung tumor cells and chemotherapy that unveiled significant insights into the role of PCPs on cancer cachexia-associated inflammation and metabolic disorders. According to various researchers (Shen et al., 2021; Li et al., 2023; Tang et al., 2023), PCPs could effectively treat cancer cachexia and muscle atrophy related to the regulation of the autophagy–lysosome and ubiquitin–proteasome systems and ATP generation. In this research, the administration dosage was established based on the standard clinical adult dose of PC outlined in the Chinese Pharmacopoeia (15 g/d). The PCP content in the PC water extract was converted to an administration dosage of 64 mg/kg/d in mice. The low-dose group was then set at half of this calculated dosage. In this study, the results implied that PCPs offer an additional therapeutic approach for cancer cachexia.

Excessive energy consumption is the most important feature in the onset and progression of cancer cachexia. Muscle and fat, as two target organs for energy storage, exhibit severe atrophy in this multifactorial metabolic syndrome, which is considered to be a hallmark of cancer cachexia. In order to visualize the disease progression of model animals with cancer cachexia, the body weight, histopathological section, and the expression of recognized markers of muscle and fat atrophy have been monitored. Atrogin-1 and MuRF1 have been regarded to encode E3 ubiquitin ligases in skeletal muscle. The breakdown of myofibrillar proteins occurs via the UPS, and the expression levels of critical regulatory proteins show consistent elevation in various models of skeletal muscle atrophy (Clavel et al., 2006). Mitochondrial dysfunction is regarded as a contributor to energy depletion and triggers protein breakdown in cancer cachexia. Muscle electron transport chain and ATP synthase are recognized as pivotal elements within mitochondrial respiratory function, closely associated with oxidative phosphorylation (OXPHOS). As features of mitochondrial dysfunction, interference with the ETC and ATP synthase reduces mitochondrial respiration and the capacity to generate ATP (Nolfi-Donegan et al., 2020). Mitochondrial COX is the final enzyme in the electron transport chain and facilitates the transfer of electrons from reduced cytochrome c to molecular oxygen, which actively pumps protons to generate the gradient utilized by ATP synthase in ATP production (Timón-Gómez et al., 2018). ATP5A1-provided gene is responsible for encoding a subunit of mitochondrial ATP synthase (Zhang et al., 2022). COXⅣ represents a nuclear-encoded isoform of COX subunit 4 (Timón-Gómez et al., 2018). Most of the mitochondrial proteins are encoded in the nucleus, synthesized as precursors in cytosolic ribosomes, and then imported into mitochondria through the translocase of the outer membrane (TOM). TOM20 serves as an initial docking site within the TOM complex, forming a common entry gate (Shiota et al., 2015). Our results were consistent with previous studies, where ATP5A, COXⅣ, and TOM20 were significantly downregulated in cachectic muscle, while cytochrome c was upregulated (Mao et al., 2021; Wei et al., 2022). High-dose PCP treatment reversed the abovementioned abnormal indexes and restored mitochondrial biogenesis such as OXPHOS and COX activity. UCP1 is a protein located in the mitochondrial membrane and carried out by brown adipocytes that are primarily responsible for facilitating adaptive thermogenesis (Petruzzelli et al., 2014). Consistent with the previous study, phenotypic detection in our experiment through IHC, HE, and WB presented an LLC-induced mice cachexia (Zhuang et al., 2016), while PCP supplementation improved muscle atrophy, muscle mitochondria dysfunction, and fat browning of the cachectic mice.

Cancer cachexia and chemotherapy create a disturbed metabolic environment that can result in diverse adverse effects on the gut microenvironment, such as intestinal epithelium, commensal bacteria, and its metabolites (Fay et al., 2017). Meanwhile, gut microenvironment dysfunction in cachexia can rapidly deteriorate the patient’s condition because it has been linked to pathways involving energy imbalance, systemic inflammation, and the disruption of the gut barrier (Rocha et al., 2023; Valdes et al., 2018). Hence, the gut and its microbiota are increasingly recognized as potential targets for cachexia therapy. In our study, it was found that high-dose PCP supplementation relieved gut microenvironment dysfunction by improving intestinal barrier integrity, reshaping the fecal gut microbiota dysbiosis, and attenuating the fecal metabolomics and SCFA disorders.

Chemotherapy affects all components of the intestinal barrier, including the mucus layer and intestinal glands (Dahlgren and Lennernäs, 2023). Tight junctions (TJs), including different types of transmembrane proteins (e.g., occludin, claudins, and zonula occludens), serve as the structural foundation for the barrier function of the intestinal epithelium (Jiang et al., 2014). The impairment of their functionality is closely linked to metabolic and inflammatory disorders (Lee et al., 2018). Alterations in gut microbiota in cancer cachexia substantially enhance the presence of Gram-negative bacteria, which, in turn, trigger pro-inflammatory responses by binding lipopolysaccharides (LPS) to toll-like receptor 4 (TLR4) (Bindels et al., 2018). Various pro-inflammatory cytokines have the capacity to disrupt the structure of TJs, thus impacting the integrity of the epithelial barrier. The elevated systemic inflammatory status is frequently observed in cachexia patients (McGovern et al., 2022). In this context, inflammatory cytokines such as TNF-α, NF-κB, IL-6, and LPS are secreted in the intestine. These cytokines disrupt the structure of TJs in the gut barrier, allowing them to enter the circulation. Consequently, this process leads to more severe systemic inflammation (Sanders, 2005). Therefore, assessing intestinal inflammation and permeability is of great value in monitoring cachectic hallmarks.

In elucidating the underlying mechanisms of PCPs in preventing gut microenvironment disruption and cachexia via the key bacteria, the diverse community of microorganisms was investigated. High-dose PCP administration can regulate the diversity and changes in microbial communities in cancer cachectic mice. At the phylum level, compared to the GCL group, PH treatment reversed the increased abundance of *Verrucomicrobiota* and *Proteobacteria*. In addition, the decrease in the abundance of *Firmicutes* and *Actinobacteria* decreased induced by the cancer cachectic model was also restored by PH treatment. At the same time, PH treatment reduced the abundance of *Desulfobacterota* and increased the abundance of *Bacteroidota*. Among them, *Bacteroidota* (Cummings and Macfarlane, 1997) participate in the metabolism of glucose and lipids in the intestine, and *Firmicutes* (Cockburn and Koropatkin, 2016) often specialize in degrading a specific group of glycans, which are the two most dominant phyla and essential for energy metabolism. Various research studies showed that the abundance of *Proteobacteria* was found to be increased in both cachectic cancer patients and cachectic C26 mice (Ubachs et al., 2021). *Proteobacteria* (Shen et al., 2017) and *Desulfobacterota* (Huang et al., 2021) can lead to an increase in intestinal endotoxin production. At the genus level, PH treatment significantly reversed the increased abundance of *Klebsiella*, *Akkermansia*, *norank_f__Desulfovibrionaceae*, *Enterococcus*, *NK4A214_group*, *Eubacterium_fissicatena_group*, *Eubacterium_nodatum_group*, and *Erysipelatoclostridium* and the decreased abundance of *Lactobacillus, Monoglobus, Ruminococcus, Odoribacter,* and *Enterorhabdus* in cachectic mice. One of the *Klebsiella* species, *Klebsiella oxytoca* (Pötgens et al., 2018), has been identified in cancer cachectic mice and has been shown to act as a gut pathobiont by altering gut barrier function. This is consistent with our experimental results. *Akkermansia* belongs to the phylum Verrucomicrobiota. Although *Akkermansia* has been regarded as a probiotic in obesity, a study involving 3,453 participants from the US found that the relative abundance of *Akkermansia* in patients with sarcopenia increased significantly (Wu and Chen, 2021), which is consistent with our experimental results. The abundance of *Enterococcus* was enriched in cachectic patients, while *Enterococcus* species have the potential to induce various purulent infections. Specifically, *Enterococcus gallolyticus* is known to lead to bacteremia and has been linked to colon cancer (Abdulamir et al., 2011; Panebianco et al., 2023; Suez et al., 2019). At the same time, *NK4A214_group*, *Norank_f_desulfovibrionaceae*, *Eubacterium_fissicatena_group*, *Erysipelatoclostridium*, and *Eubacterium_nodatum_group* show positive correlations with intestinal inflammatory reactions, respectively (Cai et al., 2022; Liu et al., 2023; Wang et al., 2023; Zheng et al., 2020). This association may be one of the reasons for the overexpression of IL-6, TNF-α, and NF-κB in the intestine of the GCL group. On the other hand, *Lactobacillus* is a widely recognized probiotic strain*.* In particular, Bindels et al. (2012) documented the prospective utilization of the *Lactobacillus* genus as an innovative therapeutic strategy for addressing certain facets of cancer cachexia. The *Monoglobus* genus is negatively associated with the onset of ulcerative colitis and represents a significant group of specialized microorganisms responsible for fermenting pectin and mannan in the human colon (Burakova et al., 2022; Kim et al., 2019). This fermentation process promotes better nutrient digestion and enhances energy utilization. *Ruminococcus* constitutes a common component of the human intestinal microbiota and is observed to be more prevalent in individuals with greater muscle mass but reduced in those with cirrhosis (Bajaj et al., 2012; Hioki et al., 2023). *Odoribacter* is capable of producing succinate, propionate, acetate, butyrate, isobutyrate, and isovalerate through the fermentation of carbohydrates *in vitro* (Göker et al., 2011). This capability may be one of the reasons for the significant increase in SCFAs observed after PH intervention in our results. Additionally, the relative abundance of *Ruminococcus* is inversely correlated with systemic inflammation in the cirrhosis model (Lakshmanan et al., 2022). The function of *Enterorhabdus* remains considerably ambiguous, with some findings indicating that it could potentially have a beneficial impact on preserving the intestinal barrier function in diabetic mice (Guo et al., 2020). In summary, cachexia modeling increases the enrichment of pathogenic bacteria, while PH administration can reverse the enrichment of those pathogenic bacteria and promote the growth of beneficial bacteria.

A growing body of evidence suggests that the microbiome, along with its metabolites, referred to as “microbial co-metabolites,” has a profound impact on various diseases (Hills et al., 2019). Using the HPLC-QE-MS/MS metabolomics platform, we conducted a comprehensive analysis of endogenous metabolites in the feces following PH administration. Our investigation revealed that PH ameliorated the chemotherapy-induced cancer cachexia model in mice. This improvement may be attributed to the increase in AAs, peptides, and analogs and the reduction in eicosanoids. The metabolic pathways of AAs undergo significant alterations in cancer cells, where AAs are utilized for both energy generation and facilitation of cell proliferation. The deprivation of AAs may intensify malnutrition and cachexia, whereas supplementation of individual AAs or combinations of essential AAs can enhance nutritional status, mitigate cachexia, and potentially enhance the patient’s responsiveness to anti-cancer treatments (Ragni et al., 2022). The branched-chain AAs such as leucine, valine, and isoleucine have been shown to enhance athletic performance (Martinho et al., 2022). Of these three AAs, leucine has been considered to play a primary regulatory role in muscle protein metabolism. Leucic acid as a metabolite of leucine has been reported to enhance muscle protein synthesis and hinder protein breakdown in both clinical and experimental studies (Sumi et al., 2021). Citrulline, an AA metabolite, has been reported to stimulate muscle protein synthesis in malnourished rats (Jourdan et al., 2015). Histamine signaling may play different roles at different stages of cachexia progression; in the early phase, increased histamine signaling, mediated by increased sympathetic tone, is associated with symptomatic loss of adipose tissue. In the late phase, histamine signaling decreases, resulting in decreased sympathetic tone and symptomatic muscle wasting (Zwickl et al., 2019). Despite the ongoing controversy surrounding the role of histamine in cachexia, some studies have reported that histamine can potentially reduce myelin basic protein loss in the spinal cord and also decrease muscle atrophy and denervation of the neuromuscular junction (Volonté et al., 2019). In addition, the PH treatment group corrected the disorder of lactic acid. Lactic acid fermentation is a process that sustains a rapid production of ATP in the absence of oxygen by glycolysis into lactic acid. Lactate serves as a versatile signaling molecule across various cells and tissues, including adipose tissue and skeletal muscles, leading to a wide range of biological effects (Brooks, 2018). These effects include increased ATP production, reduced lipolysis (Ahmed et al., 2010), and improved exercise performance (Hashimoto et al., 2006; Zhang et al., 2020). Studies conducted in mice have indicated that specific bacterial taxa can improve endurance exercise performance by enhancing various aspects of lactate metabolism (Sales and Reimer, 2023). On the other hand, eicosanoids are unsaturated C20 fatty acids that can be categorized into lipoxygenase products and prostanoids. They play crucial roles in the inflammatory process and have been implicated in the development of cancer cachexia (Ross and Fearon, 2002). For instance, prostaglandin inhibitors eliminate the muscle protein breakdown in mice experiencing cancer cachexia. These inhibitors also have the capability to avert the experimental cachectic effects induced by TNF-α and IL-1 (Kozak et al., 1997; Smith and Tisdale, 1993).

SCFAs are commonly believed to play an essential role in mediating the relationship between the gut microbiota and skeletal muscle (Ticinesi et al., 2017). Fecal levels of all SCFAs tended to be lower in cachectic cancer patients, with acetate showing a significant reduction (P < 0.05) (Ubachs et al., 2021). SCFAs are believed to influence skeletal muscle metabolism by regulating lipid, carbohydrate, and protein dynamics (Frampton et al., 2020). Butyric acids are incorporated into the tricarboxylic acid (TCA) cycle as acetyl-CoA, which serve as substrates for hepatic *de novo* lipogenesis (den Besten et al., 2013), while propanoic acids enter TCA as succinyl-CoA and act as precursors for hepatic gluconeogenesis (Boets et al., 2017). Butyric acid has been found to enhance the expression of PPAR-δ in both L6 myotubes and skeletal muscle of C57BL/6J mice *in vivo* (Gao et al., 2009). PPAR-δ is a crucial regulator of lipid and glucose metabolism, as well as skeletal muscle fiber type. Otherwise, propanoic acids and butyric acids exhibit anti-inflammatory properties that are likely mediated through the activation of FFAR2/FFAR3 or inhibition of HDAC (McLoughlin et al., 2017). Although only a few studies have discussed the molecular mechanisms of cancer cachexia related to valeric acid, branched-SCFAs (isobutyric acid and isovaleric acid), there was clinical evidence that these fatty acids are negatively associated with sarcopenia (Peng et al., 2023). In our study, the PH treatment group corrected the concentration of total SCFAs, propanoic acid, butyric acid, valeric acid, isobutyric acid, and isovaleric acid. However, when comparing the FFAR2 and PH groups, the anti-cancer cachexia effect of PH involves both modified gut microbiota and metabolites, rather than relying solely on an SCFA-dependent mechanism.

**FIGURE 8:**
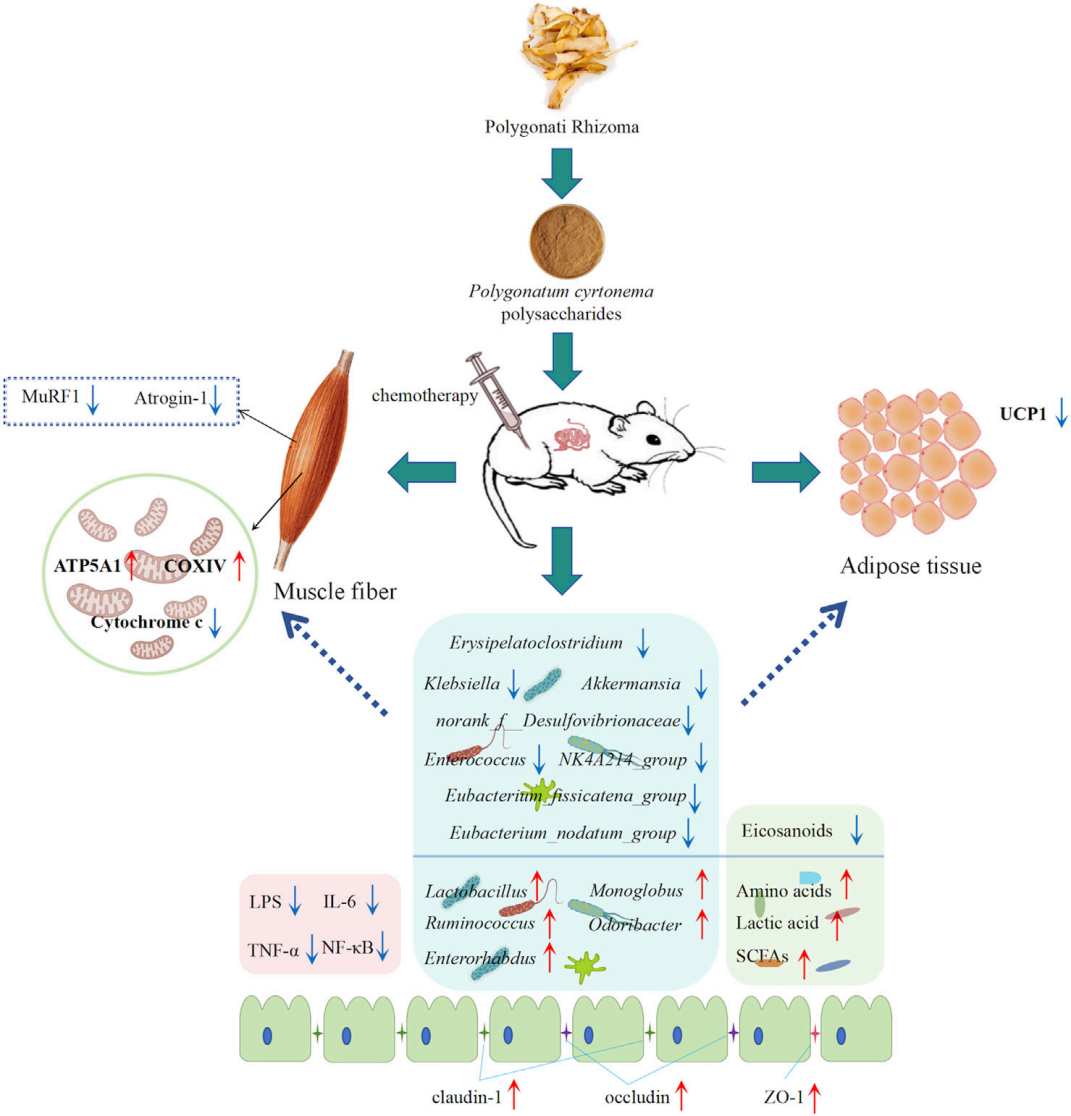
*Polygonatum cyrtonema* Hua polysaccharides improved cancer cachexia by regulating muscle fiber atrophy and adipose tissue wasting and restoring the gut microenvironment.

Our study provides new insights into anti-cancer cachexia from the perspective of intestinal microbial profiles and fecal metabolomics, which may guide the selection of natural drugs for cancer cachexia treatment (Figure 8). However, this study has limitations. First, it primarily focused on elucidating the underlying molecular mechanisms through which PCPs affect muscle atrophy and fat lipolysis. However, incorporating behavioral assessments, such as grip strength tests, voluntary wheel running activity, and evaluations of fatigue resistance, would provide a more comprehensive understanding of its therapeutic effects. Meanwhile, this study primarily focused on investigating the overall changes in gut microbiota and fecal metabolomics associated with the therapeutic effects of PCPs. Future fecal microbiota transplantation (FMT) experiments will be essential for establishing a direct causal relationship between the modulation of the gut microbiota and the observed therapeutic benefits of PCPs in chemotherapy-induced cachexia.

## 5 Conclusion

In summary, PH significantly regulated body weight loss, recovered muscle fiber atrophy and adipose tissue wasting, and attenuated gut microenvironment dysfunction in a chemotherapy-induced cancer cachectic mice model. In particular, this work reveals that PH mitigates the side effects of the cancer cachectic model by regulating gut microbiota and associated metabolic processes. The reversal of bacteria such as *Klebsiella*, *Akkermansia*, *norank_f__Desulfovibrionaceae*, *Enterococcus*, *NK4A214_group*, *Eubacterium_fissicatena_group*, *Eubacterium_nodatum_group*, *Erysipelatoclostridium* and *Lactobacillus, Monoglobus*, *Ruminococcus*, *Odoribacter, Enterorhabdus*, as well as metabolites like AAs, eicosanoids, lactic acid, and SCFAs, may be related to the therapeutic effects of PH.

## Data Availability

The datasets presented in this study can be found in online repositories. The 16S rRNA sequencing data have been uploaded to NCBI under Bioproject ID: PRJNA1064893.
