# Supplementary material for: Polygonatum cyrtonema Hua polysaccharides alleviate muscle atrophy and fat lipolysis by regulating the gut microenvironment in chemotherapy-induced cachexia
Source: Front Pharmacol. 2025 Mar 10;16:1503785. doi: 10.3389/fphar.2025.1503785 (PMC11931129; doi:10.3389/fphar.2025.1503785)
Supplement: Supplementary file 1 [file Table1.docx]

Supplementary Material

# Supplementary Tables

**Table. S1.** The significant different metabolites between CTRL and GCL group. Variable importance in the projection (VIP) was obtained from OPLS-DA with a threshold of 1.0. P value was calculated from student's t-test. Fold change(FC) was calculated from the arithmetic mean values between CTRL and GCL group with a thresold of 1.2 or 0.83.

| **No.** | **Rt** | **Metabolite ID** | **m/z** | **Detection mode** | **HMDB_ID** | **Identification** | **FC** | ***P value*** |
| --- | --- | --- | --- | --- | --- | --- | --- | --- |
| 1 | 0.554216667 | metab_8748 | 261.0299286 | pos | HMDB0014672 | Cyclophosphamide | 1.217 | 0.0002452 |
| 2 | 0.562166667 | metab_8428 | 170.0251772 | pos | HMDB0245056;HMDB0243770;HMDB0246657 | 2-Methylthiazolidine-4-carboxylic acid | 1.218 | 0.0002663 |
| 3 | 0.58565 | metab_8353 | 112.0870178 | pos | HMDB0000870 | Histamine | 1.2114 | 0.0009556 |
| 4 | 0.596083333 | metab_8970 | 249.049507 | neg | HMDB0245528 | 1-((2S,4S,5R)-4-Hydroxy-5-(hydroxymethyl)tetrahydrofuran-2-yl)pyrimidine-2,4(1H,3H)-dione | 1.3642 | 0.0002772 |
| 5 | 0.6196 | metab_15202 | 202.0201322 | neg | HMDB0002135 | S-(3-Oxo-3-carboxy-n-propyl)cysteine | 1.4816 | 0.000008286 |
| 6 | 0.672516667 | metab_1545 | 232.1399104 | pos | HMDB0034326 | L-erythro-4-Hydroxyarginine | 1.2207 | 0.02604 |
| 7 | 0.689433333 | metab_15002 | 272.0545076 | neg | HMDB0252080 | Ethylmethylhydroxypyridine succinate | 0.7212 | 8.898E-07 |
| 8 | 0.705033333 | metab_14993 | 189.0873593 | neg | HMDB0031412 | 2-Amino-4-[(2-hydroxy-1-oxopropyl)amino]butanoic acid | 1.208 | 0.0002193 |
| 9 | 0.767166667 | metab_15793 | 323.0282427 | neg | HMDB0000288 | Uridine 5'-monophosphate | 0.702 | 0.02325 |
| 10 | 0.774983333 | metab_14816 | 233.0298903 | neg | HMDB0059808 | Acetyl citrate | 0.8048 | 0.003488 |
| 11 | 0.790583333 | metab_14791 | 160.0243101 | neg | HMDB0254310 | MALEAMIC ACID | 1.2562 | 0.00252 |
| 12 | 0.821766667 | metab_8787 | 166.9953547 | neg | HMDB0248224 | Alpha-Ketoglutarate cyanohydrin | 0.6274 | 0.03077 |
| 13 | 0.985733333 | metab_15860 | 269.0875297 | neg | HMDB0000197;HMDB0000190;HMDB0001311 | Lactic Acid | 1.3166 | 0.001444 |
| 14 | 1.032983333 | metab_7715 | 154.0971464 | pos | HMDB0013253 | Nomega-Acetylhistamine | 1.4366 | 0.0001292 |
| 15 | 1.0401 | metab_9227 | 171.0290789 | neg | HMDB0304122 | 3-Dehydroshikimate | 0.7793 | 0.0003877 |
| 16 | 1.055583333 | metab_14681 | 346.1250106 | neg | HMDB0034363 | Monocrotaline | 0.8322 | 0.01471 |
| 17 | 1.055583333 | metab_14685 | 236.0229776 | neg | HMDB0244750 | 2,4-Thiazolidinedicarboxylic acid, 2-methyl- | 0.8324 | 0.000492 |
| 18 | 1.086583333 | metab_14661 | 319.1142751 | neg | HMDB0257719 | N-[[(2S,3S,4R,5R)-5-(6-Aminopurin-9-yl)-3,4-dihydroxyoxolan-2-yl]-hydroxymethyl]-N-ethylformamide | 0.762 | 0.000008194 |
| 19 | 1.15645 | metab_9287 | 323.0282071 | neg | HMDB0000288 | Uridine-5'-Monophosphate | 0.6329 | 0.01406 |
| 20 | 1.211183333 | metab_9309 | 274.0566168 | neg | HMDB0304164 | 4,5-seco-dopa | 0.7894 | 0.00715 |
| 21 | 1.312383333 | metab_14561 | 346.0553146 | neg | HMDB0003540 | Adenosine 3'-monophosphate | 0.7178 | 0.02174 |
| 22 | 1.34425 | metab_9354 | 216.0985167 | neg | HMDB0000856 | N-alpha-Acetyl-L-citrulline | 1.3778 | 0.00001983 |
| 23 | 1.4219 | metab_9365 | 267.1459415 | neg | HMDB0028889;HMDB0253179 | Histidinyl-Leucine | 1.2336 | 0.03293 |
| 24 | 1.55335 | metab_1882 | 220.1538884 | pos | HMDB0059708 | 2-Ethylsuberic acid | 1.2689 | 0.0007543 |
| 25 | 1.7099 | metab_14464 | 293.0878243 | neg | HMDB0303141 | Tuliposide B | 1.3882 | 0.0142 |
| 26 | 1.717733333 | metab_9436 | 321.0489102 | neg | HMDB0001227 | 5'-Thymidylic Acid | 0.6711 | 0.0002244 |
| 27 | 1.741283333 | metab_9441 | 289.1402052 | neg | HMDB0242138 | (2R)-2-Amino-6-[[(3S,4R,5R)-3,4,5,6-tetrahydroxy-2-oxohexyl]amino]hexanoic acid | 1.2987 | 0.000006357 |
| 28 | 1.749116667 | metab_14454 | 205.0646981 | neg | HMDB0028973 | Methionyl-Glycine | 1.5048 | 0.01024 |
| 29 | 1.7634 | metab_7400 | 207.0794517 | pos | HMDB0028847 | Glycyl-Methionine | 1.2456 | 0.001799 |
| 30 | 1.779183333 | metab_1915 | 93.07016707 | pos | HMDB0250648 | Cyclohexa-2,4-dienylmethanol | 0.8121 | 0.00249 |
| 31 | 1.7949 | metab_1924 | 431.2013868 | pos | HMDB0036864 | Anhydrocinnzeylanine | 0.7814 | 0.0003091 |
| 32 | 1.83455 | metab_7376 | 198.0580989 | pos | HMDB0257715 | (2R)-2-Acetamido-6-hydroxy-2-(sulfanylmethyl)hex-3-enoic acid | 0.8252 | 0.006572 |
| 33 | 1.835433333 | metab_14428 | 344.0397235 | neg | HMDB0001314 | 3',5'-Cyclic GMP | 0.7201 | 0.00007184 |
| 34 | 1.890133333 | metab_7268 | 292.1649758 | pos | HMDB0029876 | Paucine | 1.2133 | 0.0007339 |
| 35 | 1.90595 | metab_14411 | 575.1492685 | neg | HMDB0035872 | Oleuropein | 1.2887 | 0.007151 |
| 36 | 1.921583333 | metab_15841 | 346.0553928 | neg | HMDB0000045;HMDB0003540;HMDB0011617 | Adenosine 2'-phosphate | 0.3251 | 0.04668 |
| 37 | 1.93005 | metab_14335 | 211.0007273 | neg | HMDB0000618 | D-Ribulose 5-Phosphate | 1.3675 | 0.002452 |
| 38 | 1.945716667 | metab_14287 | 335.0609136 | neg | HMDB0252788 | Glucose pyruvate lactate | 0.2957 | 0.002757 |
| 39 | 2.040433333 | metab_14259 | 293.0876618 | neg | HMDB0302563 | Arabinogalactose | 1.3632 | 0.001194 |
| 40 | 2.122983333 | metab_7139 | 251.1496581 | pos | HMDB0028888 | Histidylisoleucine | 1.5476 | 0.03341 |
| 41 | 2.146916667 | metab_8728 | 434.1882107 | pos | HMDB0249379 | Brexpiprazole | 1.2207 | 0.000000026 |
| 42 | 2.28965 | metab_9587 | 292.1663608 | neg | HMDB0304786;HMDB0028958 | Lysylphenylalanine | 1.2955 | 0.01157 |
| 43 | 2.346116667 | metab_9596 | 243.1458312 | neg | HMDB0252056 | Ethyl N2-acetyl-L-argininate | 1.3002 | 0.0008133 |
| 44 | 2.410616667 | metab_3287 | 347.1443007 | pos | HMDB0060494 | N-Acetylmuramoyl-Ala | 1.2089 | 0.001521 |
| 45 | 2.45125 | metab_7039 | 168.0287511 | pos | HMDB0000232 | Quinolinic Acid | 1.3886 | 0.00001638 |
| 46 | 2.6006 | metab_2133 | 178.0529433 | pos | HMDB0001015 | N-Formyl-L-methionine | 0.7244 | 0.00004806 |
| 47 | 2.731683333 | metab_9634 | 279.0983115 | neg | HMDB0011167 | L-beta-aspartyl-L-phenylalanine | 1.2731 | 0.004497 |
| 48 | 2.839966667 | metab_6691 | 170.1536323 | pos | HMDB0304704 | Lupinine | 0.828 | 0.02357 |
| 49 | 2.944466667 | metab_349 | 539.2075528 | pos | HMDB0039230 | Citrusin A | 0.7105 | 0.002567 |
| 50 | 2.944466667 | metab_6603 | 521.1968027 | pos | HMDB0015152 | Minocycline | 0.8181 | 0.007065 |
| 51 | 2.984483333 | metab_6570 | 334.160292 | pos | - | Ser Asp Ile | 0.6752 | 0.00122 |
| 52 | 3.040916667 | metab_9681 | 157.1337662 | neg | HMDB0255016 | N-Acetyl-1,6-diaminohexane | 1.3736 | 0.02502 |
| 53 | 3.083216667 | metab_9700 | 386.1202185 | neg | HMDB0062585 | (3R,5S)-1-pyrroline-3-hydroxy-5-carboxylic Acid | 0.7394 | 0.009049 |
| 54 | 3.09715 | metab_14080 | 299.0629124 | neg | HMDB0029920 | Beta-D-3-Ribofuranosyluric acid | 0.7958 | 0.0008976 |
| 55 | 3.09715 | metab_9093 | 309.0537681 | neg | HMDB0251268 | Difluorodeoxyuridine | 0.6631 | 6.091E-10 |
| 56 | 3.14825 | metab_14060 | 247.1119354 | neg | HMDB0259742;HMDB0029133 | Valylmethionine | 1.2098 | 0.001337 |
| 57 | 3.160433333 | metab_6419 | 466.1601408 | pos | HMDB0253989 | Latrunculin a | 1.3636 | 0.000002915 |
| 58 | 3.2019 | metab_9744 | 653.2522178 | neg | HMDB0033909 | 2'-Deoxymugineic acid | 1.2495 | 0.01427 |
| 59 | 3.309566667 | metab_2463 | 263.0480256 | pos | HMDB0253636 | Isoflavanone | 0.3535 | 0.005005 |
| 60 | 3.317533333 | metab_6288 | 602.3133089 | pos | HMDB0247956;HMDB0031448 | Acolongifloriside K | 0.7504 | 0.02579 |
| 61 | 3.42055 | metab_2502 | 385.1055827 | pos | HMDB0247465 | 8-Prenylnaringenin | 0.7581 | 2.904E-09 |
| 62 | 3.505016667 | metab_13879 | 218.0123589 | neg | HMDB0304940 | 2-Aminobenzenesulfonic acid | 1.2148 | 0.00002252 |
| 63 | 3.525733333 | metab_6166 | 311.1703995 | pos | HMDB0029066 | Threonyllysine | 2.3295 | 0.01262 |
| 64 | 3.544683333 | metab_13855 | 431.1124071 | neg | HMDB0035828 | Lactupicrin | 0.8113 | 0.00003107 |
| 65 | 3.544683333 | metab_13856 | 366.1050732 | neg | HMDB0006832 | S-Glutaryldihydrolipoamide | 0.8283 | 0.01411 |
| 66 | 3.557483333 | metab_9905 | 519.1828201 | neg | HMDB0060711 | 2-Amino-5-benzoylbenzimidazole | 0.7466 | 0.0007293 |
| 67 | 3.57075 | metab_13840 | 302.1624283 | neg | HMDB0028989 | Phenylalanylarginine | 1.2899 | 0.002196 |
| 68 | 3.589733333 | metab_6128 | 385.1056048 | pos | HMDB0034214 | 5-Deoxykievitone | 0.631 | 1.177E-07 |
| 69 | 3.598233333 | metab_6114 | 495.2957769 | pos | HMDB0060527 | Kukoamine D | 1.2551 | 0.01768 |
| 70 | 3.614216667 | metab_2567 | 415.1161904 | pos | HMDB0060328 | 1-Nitro-5,6-dihydroxy-dihydronaphthalene | 0.7835 | 0.0007864 |
| 71 | 3.655483333 | metab_13792 | 340.0594638 | neg | HMDB0013854 | N4-Acetylsulfamethoxazole | 0.166 | 4.099E-07 |
| 72 | 3.65995 | metab_9949 | 677.2522148 | neg | HMDB0247526 | N(G)-Nitroarginine-4-nitroanilide | 0.7811 | 0.0005833 |
| 73 | 3.662233333 | metab_6092 | 421.2106065 | pos | HMDB0301837 | 12-Hydroxyjasmonic acid glucoside | 0.8191 | 0.0007936 |
| 74 | 3.676233333 | metab_9957 | 341.1730613 | neg | HMDB0029077 | Tryptophyl-Arginine | 1.2704 | 0.002601 |
| 75 | 3.70235 | metab_9963 | 275.1245063 | neg | HMDB0012880 | Acetamidopropanal | 3.8425 | 0.003943 |
| 76 | 3.70235 | metab_13770 | 302.0700985 | neg | HMDB0256775 | Prinomide | 1.4388 | 0.0003326 |
| 77 | 3.715266667 | metab_9970 | 246.0074683 | neg | HMDB0247292 | 7,8-Dichloro-1,2,3,4-tetrahydroisoquinoline | 1.3243 | 0.00603 |
| 78 | 3.746816667 | metab_13735 | 394.1978139 | neg | HMDB0252886 | Glycyllysylarginine | 0.7763 | 0.0002981 |
| 79 | 3.750733333 | metab_6041 | 403.2694782 | pos | HMDB0003252 | Thromboxane B2 | 0.4859 | 4.069E-07 |
| 80 | 3.763133333 | metab_13724 | 354.0253276 | neg | HMDB0006824;HMDB0304239 | 7,8-dihydroneopterin 3'-phosphate | 0.7408 | 0.0003402 |
| 81 | 3.767383333 | metab_8743 | 153.1383954 | pos | HMDB0014328;HMDB0015516;HMDB0012128 | Amphetamine | 0.8162 | 0.02678 |
| 82 | 3.801416667 | metab_13701 | 135.0442034 | neg | HMDB0000209 | Phenylacetic Acid | 1.2348 | 0.000007077 |
| 83 | 3.8147 | metab_10018 | 376.1872824 | neg | HMDB0061099 | 5-hydroxy saxagliptin | 0.8301 | 0.003487 |
| 84 | 3.917716667 | metab_10060 | 351.1194226 | neg | HMDB0001013 | Cotinine glucuronide | 1.2865 | 0.00124 |
| 85 | 4.008233333 | metab_13601 | 383.0914695 | neg | HMDB0304360 | Gibberellin A29-catabolite | 0.8144 | 6.723E-08 |
| 86 | 4.017083333 | metab_5922 | 437.2023648 | pos | HMDB0245144 | 2-Hydroxy-3-methoxy-6beta-naltrexol | 1.4006 | 0.00002028 |
| 87 | 4.0299 | metab_10104 | 220.0645036 | neg | HMDB0258091 | (2R)-2-Acetamido-5-hydroxy-2-(sulfanylmethyl)pentanoic acid | 1.2079 | 0.001765 |
| 88 | 4.033083333 | metab_2683 | 173.1070933 | pos | HMDB0245222 | 2-Methyl-5-hydroxytryptamine | 0.77 | 0.02947 |
| 89 | 4.037733333 | metab_13589 | 275.0229219 | neg | HMDB0251585 | Doqualast | 1.4385 | 0.0005354 |
| 90 | 4.049083333 | metab_2686 | 407.2645478 | pos | - | Leu Lys Phe | 0.8224 | 0.04114 |
| 91 | 4.049083333 | metab_5909 | 439.2215575 | pos | HMDB0259814 | Vindoline | 0.8119 | 0.011 |
| 92 | 4.0883 | metab_10126 | 254.1142385 | neg | HMDB0041809 | 4-(Methylnitrosamino)-1-(3-pyridyl)-1-butanol | 0.7663 | 0.01774 |
| 93 | 4.1226 | metab_13559 | 325.1190588 | neg | HMDB0060486;HMDB0034666 | Mandelonitrile | 3.8717 | 0.0007748 |
| 94 | 4.1616 | metab_13536 | 252.0873888 | neg | HMDB0002042;HMDB0000860 | 3-Phenylpropionylglycine | 1.2085 | 0.00007816 |
| 95 | 4.161783333 | metab_5857 | 405.2853698 | pos | HMDB0256669 | 6-Amino-N-[6-keto-6-(6-ketohexylamino)hexyl]hexanamide | 0.6854 | 0.002028 |
| 96 | 4.169433333 | metab_10160 | 383.0914578 | neg | HMDB0041372 | Alectrol | 0.8042 | 2.097E-08 |
| 97 | 4.20245 | metab_2730 | 311.102782 | pos | HMDB0060832 | Meta-O-Dealkylated flecainide lactam | 1.3099 | 0.0002238 |
| 98 | 4.22645 | metab_5841 | 315.1947657 | pos | HMDB0003034 | Prostaglandin D3 | 0.8082 | 0.000001698 |
| 99 | 4.242466667 | metab_711 | 333.2057835 | pos | HMDB0004242;HMDB0244347 | 11-Dehydro-thromboxane B2 | 0.7826 | 0.000000617 |
| 100 | 4.268466667 | metab_13491 | 213.9632141 | neg | - | 2-Benzothiazolsulfonic Acid | 1.261 | 0.003005 |
| 101 | 4.283133333 | metab_5813 | 604.3217901 | pos | HMDB0253595 | Isbogrel | 1.2263 | 0.004222 |
| 102 | 4.290983333 | metab_13481 | 483.1549115 | neg | HMDB0060557 | Beta-(2-Methoxyphenoxy)-lactic acid | 1.419 | 0.000054 |
| 103 | 4.30565 | metab_10200 | 305.069742 | neg | HMDB0251458 | Diphenylhydantoic acid | 0.8118 | 0.002224 |
| 104 | 4.307133333 | metab_731 | 585.3022982 | pos | - | Lys Ala Thr Glu His | 0.7354 | 0.000009274 |
| 105 | 4.357583333 | metab_10211 | 385.1071808 | neg | HMDB0303458 | Gibberellin A29 | 0.7826 | 0.00002107 |
| 106 | 4.364483333 | metab_13458 | 314.1144397 | neg | HMDB0028830 | Glutamyltryptophan | 2.1607 | 0.002396 |
| 107 | 4.411766667 | metab_751 | 421.2111237 | pos | HMDB0015019 | Quinapril | 0.7106 | 0.004789 |
| 108 | 4.415183333 | metab_13443 | 311.0895044 | neg | HMDB0256914 | Pteroic acid | 1.3767 | 0.0005032 |
| 109 | 4.468283333 | metab_2783 | 423.2270252 | pos | HMDB0247885 | 3-[2-[[(1S,2R,3S)-3-[4-(Pentylcarbamoyl)-1,3-oxazol-2-yl]-7-oxabicyclo[2.2.1]heptan-2-yl]methyl]phenyl]propanoic acid | 0.8131 | 0.00476 |
| 110 | 4.482683333 | metab_13422 | 504.177489 | neg | HMDB0251123 | Diacylglycerol kinase inhibitor i | 1.2932 | 0.00004576 |
| 111 | 4.4961 | metab_10247 | 395.2069442 | neg | HMDB0247474;HMDB0002664 | Prostaglandin E3 | 0.7486 | 0.0001877 |
| 112 | 4.524283333 | metab_5717 | 284.1041865 | pos | HMDB0029079 | Tryptophyl-Aspartate | 0.7633 | 0.0439 |
| 113 | 4.527183333 | metab_13406 | 305.1868327 | neg | HMDB0015070 | Oxymetazoline | 0.8224 | 0.02534 |
| 114 | 4.548133333 | metab_10261 | 356.11943 | neg | HMDB0061011 | N-desmethylalmotriptan | 1.7208 | 0.001494 |
| 115 | 4.560633333 | metab_13396 | 340.2237427 | neg | HMDB0251473 | DIPROTIN A | 0.8259 | 0.002885 |
| 116 | 4.564166667 | metab_10266 | 381.2278519 | neg | HMDB0039171 | 9alpha-(3-Methylbutanoyloxy)-4S-hydroxy-10(14)-oplopen-3-one | 0.6737 | 0.000000266 |
| 117 | 4.584333333 | metab_13387 | 330.1666289 | neg | - | N-Acetylleucylalanylserine | 1.2455 | 0.001608 |
| 118 | 4.66065 | metab_5658 | 234.1484486 | pos | HMDB0040833 | 3-[4-Hydroxy-3-(3-methyl-2-butenyl)phenyl]-2-propenal | 1.2873 | 0.000003425 |
| 119 | 4.708666667 | metab_793 | 315.1948071 | pos | HMDB0062410 | 5,6-epoxy,18R-HEPE | 0.6818 | 4.751E-08 |
| 120 | 4.7226 | metab_10310 | 393.1988981 | neg | HMDB0034365 | L-Theanine | 0.8147 | 0.007629 |
| 121 | 4.7542 | metab_13336 | 259.1547558 | neg | HMDB0035028 | (x)-2-Heptanol glucoside | 1.8363 | 0.00006018 |
| 122 | 4.762033333 | metab_10321 | 395.2069259 | neg | HMDB0062297 | 9S-hydroxy-11,15-dioxo-5Z,13E-prostadienoic acid | 0.7919 | 0.00001015 |
| 123 | 4.762033333 | metab_10320 | 317.1240593 | neg | HMDB0033663 | Decarbamoylneosaxitoxin | 1.3615 | 0.0001622 |
| 124 | 4.769383333 | metab_10323 | 385.1070765 | neg | HMDB0039062 | Triphasiol | 0.3678 | 0.0003354 |
| 125 | 4.804683333 | metab_2858 | 238.133429 | pos | HMDB0249390 | 2-((4,5-Dihydro-1H-imidazol-2-yl)methyl)-2,3-dihydro-1-methyl-1H-isoindole | 0.5199 | 0.00579 |
| 126 | 4.828666667 | metab_8759 | 331.2004591 | pos | HMDB0014568;HMDB0242263;HMDB0003573 | Hyoscyamine | 0.7792 | 0.004829 |
| 127 | 4.83715 | metab_10336 | 257.1295402 | neg | HMDB0038982 | 4-Nonylphenol | 0.8233 | 0.00001753 |
| 128 | 5.037783333 | metab_13264 | 426.1765082 | neg | HMDB0255595 | Nifekalant | 0.5162 | 2.242E-10 |
| 129 | 5.037783333 | metab_13265 | 363.1807742 | neg | HMDB0006709 | Ubiquinone-2 | 0.7619 | 8.717E-08 |
| 130 | 5.045516667 | metab_13262 | 263.1496464 | neg | HMDB0000665 | Leucinic Acid | 1.4302 | 0.0001062 |
| 131 | 5.045516667 | metab_13915 | 199.0580693 | neg | HMDB0242656 | (2S,3R,4S,5R,6R)-6-Ethyloxane-2,3,4,5-tetrol | 1.2179 | 0.00001496 |
| 132 | 5.052383333 | metab_10388 | 271.135689 | neg | HMDB0032223 | Diisopentyl thiomalate | 1.6378 | 0.002048 |
| 133 | 5.052383333 | metab_13257 | 285.1317898 | neg | HMDB0244804 | 1,4,7,10,13,16-Hexaoxacyclooctadecane | 1.3916 | 0.0001363 |
| 134 | 5.052383333 | metab_13258 | 261.0289379 | neg | HMDB0014787 | Stavudine | 1.2394 | 0.00004789 |
| 135 | 5.061333333 | metab_5511 | 450.2454395 | pos | HMDB0036836 | Cascarillin | 1.2397 | 0.0000918 |
| 136 | 5.06565 | metab_13251 | 275.1035725 | neg | HMDB0000594 | Gamma-Glutamylphenylalanine | 0.8117 | 0.002137 |
| 137 | 5.0933 | metab_5501 | 358.1541519 | pos | HMDB0259316 | Tubastatin A | 0.7098 | 0.0006334 |
| 138 | 5.11095 | metab_10401 | 393.1925658 | neg | HMDB0303454 | Gibberellin A14 | 0.7967 | 0.0001303 |
| 139 | 5.1659 | metab_2939 | 687.2680593 | pos | HMDB0251605 | (D-Pen2,D-Pen5)-Enkephalin | 0.7144 | 0.000003594 |
| 140 | 5.183966667 | metab_10418 | 381.0757949 | neg | HMDB0035029 | Dehydrocyanaropicrin | 0.7066 | 0.0004569 |
| 141 | 5.2041 | metab_10426 | 365.1964748 | neg | HMDB0006059 | 20-carboxy-LTB4 | 0.8229 | 2.709E-08 |
| 142 | 5.238533333 | metab_5455 | 477.2456963 | pos | HMDB0062632 | 1-O-all-trans-retinoyl-beta-glucuronic Acid | 0.7893 | 0.0001536 |
| 143 | 5.238533333 | metab_5456 | 336.1655824 | pos | HMDB0304396;HMDB0257214 | Riboprine | 0.2328 | 0.002785 |
| 144 | 5.271116667 | metab_5446 | 397.2111041 | pos | HMDB0259448 | 8H-Purin-8-one, 7-(2-butynyl)-7,9-dihydro-9-(6-methoxy-3-pyridinyl)-6-(1-piperazinyl)- | 0.822 | 0.002471 |
| 145 | 5.27865 | metab_13178 | 392.1823796 | neg | - | Glu-val-phe | 0.7779 | 0.0001859 |
| 146 | 5.302183333 | metab_14102 | 147.0442423 | neg | HMDB0000779 | 3-Phenyllactic Acid | 1.2206 | 0.000008732 |
| 147 | 5.327 | metab_2968 | 283.2009366 | pos | HMDB0038588 | Dioscoretine | 1.5138 | 0.002329 |
| 148 | 5.334966667 | metab_5420 | 469.2758334 | pos | HMDB0010359 | 17-Hydroxyandrostane-3-glucuronide | 1.2096 | 0.003736 |
| 149 | 5.374383333 | metab_10476 | 285.1450731 | neg | HMDB0015407 | Pirbuterol | 0.5142 | 0.03666 |
| 150 | 5.381266667 | metab_13144 | 361.1653653 | neg | HMDB0304362 | Gibberellin A36 | 0.6516 | 1.397E-07 |
| 151 | 5.41435 | metab_10488 | 202.1078303 | neg | HMDB0242158 | 1-Carboxyethylisoleucine | 1.2014 | 0.0002743 |
| 152 | 5.431533333 | metab_5397 | 484.2551186 | pos | HMDB0250778 | D-Name | 0.7664 | 0.03529 |
| 153 | 5.5274 | metab_5373 | 639.301549 | pos | HMDB0266594 | PA(20:4(6E,8Z,11Z,14Z)-OH(5S)/8:0) | 0.8065 | 0.000007668 |
| 154 | 5.5354 | metab_2997 | 370.1575549 | pos | HMDB0257991 | (2S)-2-Amino-3-[(2S,3R)-2-amino-3-[(2S)-2-amino-3-(4-hydroxyphenyl)propanoyl]oxybutanoyl]oxypropanoic acid | 0.7471 | 0.00000131 |
| 155 | 5.570033333 | metab_13098 | 381.1122756 | neg | HMDB0304361 | Gibberellin A34-catabolite | 0.716 | 0.00009786 |
| 156 | 5.679416667 | metab_5325 | 498.2225969 | pos | HMDB0036592 | Validamycin A | 0.6642 | 0.000006028 |
| 157 | 5.687416667 | metab_5318 | 459.2223069 | pos | HMDB0247652 | Gemfibrozil 1-O-beta-Glucuronide | 0.8027 | 0.001804 |
| 158 | 5.733 | metab_13049 | 371.1275836 | neg | HMDB0014507 | Clozapine | 0.7804 | 0.0001525 |
| 159 | 5.74815 | metab_13046 | 323.1603636 | neg | HMDB0255924 | P-Hydroxyubenimex | 0.8134 | 0.000009969 |
| 160 | 5.767416667 | metab_3038 | 611.3426371 | pos | HMDB0033210 | Lyciumoside VIII | 0.8322 | 0.0001197 |
| 161 | 5.813566667 | metab_13027 | 271.061057 | neg | HMDB0002670 | Naringenin | 0.8191 | 0.01564 |
| 162 | 5.815433333 | metab_1398 | 637.3203804 | pos | - | Imiprothrin | 0.7813 | 9.14E-08 |
| 163 | 5.823433333 | metab_5280 | 432.2485203 | pos | HMDB0014822 | Tobramycin | 0.8006 | 0.00003081 |
| 164 | 5.830333333 | metab_10592 | 301.1763484 | neg | HMDB0257190 | Arginylphenylalaninamide | 1.3218 | 0.00002237 |
| 165 | 5.883816667 | metab_10610 | 321.0435141 | neg | HMDB0256138 | Parylene C | 1.514 | 1.255E-07 |
| 166 | 5.883816667 | metab_15845 | 345.1344973 | neg | HMDB0036423 | Cynaropicrin | 1.2295 | 0.00001649 |
| 167 | 5.89165 | metab_12986 | 377.0698235 | neg | HMDB0245160 | 2H-1,4-Benzodiazepin-2-one, 1,3-dihydro-7-chloro-5-(2-fluorophenyl)-1-(2-hydroxyethyl)- | 2.5829 | 0.00003979 |
| 168 | 5.895283333 | metab_5247 | 637.2972471 | pos | HMDB0015129;HMDB0242630 | Neomycin sulfate | 0.7827 | 0.000133 |
| 169 | 5.899483333 | metab_10623 | 259.1451136 | neg | HMDB0094673 | Cyclo(Leu-Phe) | 0.643 | 0.001423 |
| 170 | 5.911266667 | metab_5242 | 371.111799 | pos | HMDB0303169 | Astringin | 1.412 | 3.526E-07 |
| 171 | 5.939316667 | metab_12925 | 439.1865472 | neg | HMDB0254292 | Macrolactin-A | 1.2063 | 0.001862 |
| 172 | 5.951266667 | metab_981 | 661.3190612 | pos | HMDB0241792 | (2S)-2-Hydroxy-2-(propan-2-yl)butanedioylcarnitine | 0.7422 | 1.757E-07 |
| 173 | 5.962816667 | metab_10687 | 549.2711362 | neg | HMDB0246702 | Phorbol-12,13-dibutyrate | 1.5286 | 0.0003217 |
| 174 | 5.962816667 | metab_12897 | 544.3384678 | neg | HMDB0242389 | Cholylhistidine | 0.7799 | 0.002506 |
| 175 | 5.975266667 | metab_5182 | 500.1938702 | pos | HMDB0041222 | A-L-Fucopyranosyl-(1->2)-b-D-galactopyranosyl-(1->2)-D-xylose | 0.4072 | 0.0002066 |
| 176 | 5.983266667 | metab_5174 | 606.3263153 | pos | HMDB0302594 | PL | 0.769 | 0.000183 |
| 177 | 5.999266667 | metab_3120 | 608.3423175 | pos | HMDB0249736 | Methyl 6-[(3S,6S,9S,12R)-3-butan-2-yl-6-[(1-methoxyindol-3-yl)methyl]-2,5,8,11-tetraoxo-1,4,7,10-tetrazabicyclo[10.4.0]hexadecan-9-yl]hexanoate | 0.6492 | 0.000306 |
| 178 | 6.007266667 | metab_5157 | 319.2368075 | pos | HMDB0242342 | (1R,9R,13R)-1,13-Dimethyl-10-[[(2R)-oxolan-2-yl]methyl]-10-azatricyclo[7.3.1.02,7]trideca-2(7),3,5-trien-4-ol | 1.3199 | 0.000001209 |
| 179 | 6.023266667 | metab_3134 | 498.2225367 | pos | - | Panosialin A | 0.7851 | 0.000001838 |
| 180 | 6.057383333 | metab_12760 | 564.365157 | neg | HMDB0014866 | Rocuronium | 0.6802 | 0.00002725 |
| 181 | 6.065216667 | metab_12750 | 494.3115776 | neg | HMDB0242414;HMDB0000631 | Deoxycholylglycine | 0.8063 | 3.002E-08 |
| 182 | 6.073016667 | metab_10786 | 271.133448 | neg | HMDB0030917 | 1-Hydroxyacorenone | 1.2076 | 0.00002545 |
| 183 | 6.08085 | metab_10793 | 326.0159794 | neg | HMDB0248387 | Anb-nos | 1.2421 | 0.0001701 |
| 184 | 6.103266667 | metab_1028 | 482.2276689 | pos | HMDB0248925 | Beclomethasone 17-monopropionate | 0.8018 | 0.00003056 |
| 185 | 6.104366667 | metab_10809 | 267.0297361 | neg | HMDB0002326 | Coumestrol | 1.2232 | 6.358E-07 |
| 186 | 6.112166667 | metab_12695 | 278.1395144 | neg | HMDB0034684 | Thalictroidine | 1.3019 | 0.00003618 |
| 187 | 6.12 | metab_10830 | 973.4659285 | neg | HMDB0278358 | PI(5-iso PGF2VI/22:6(4Z,7Z,10Z,13Z,16Z,19Z)) | 0.7517 | 0.0001029 |
| 188 | 6.12 | metab_12684 | 909.5041696 | neg | HMDB0285619 | PC(LTE4/14:0) | 0.7287 | 4.803E-07 |
| 189 | 6.151283333 | metab_5000 | 639.283025 | pos | HMDB0259343 | Tyr-ile-gly-ser-arg | 0.6817 | 0.003928 |
| 190 | 6.159283333 | metab_4993 | 611.3417898 | pos | HMDB0037793 | (3S,5R,6R,6'S)-6,7-Didehydro-5,6-dihydro-3,5,6'-trihydroxy-13,14,20-trinor-3'-oxo-beta,epsilon-caroten-19',11'-olide 3-acetate | 0.8245 | 0.00009227 |
| 191 | 6.159283333 | metab_4995 | 468.36714 | pos | HMDB0060137 | 3alpha,7alpha,12alpha,25-Tetrahydroxy-5beta-cholestane-24-one | 0.8274 | 5.706E-09 |
| 192 | 6.159283333 | metab_4996 | 425.2501101 | pos | HMDB0252353 | Fluocortolone Pivalate | 1.2159 | 0.000006871 |
| 193 | 6.167266667 | metab_4986 | 349.2840169 | pos | HMDB0256924 | Pumiliotoxin A | 1.3373 | 0.00000517 |
| 194 | 6.18195 | metab_10863 | 829.5472555 | neg | HMDB0039019 | 3-Hydroxy-10'-apo-b,y-carotenal | 0.7941 | 3.725E-07 |
| 195 | 6.191283333 | metab_4955 | 549.3994006 | pos | HMDB0249169 | Bevirimat | 0.7481 | 0.00005085 |
| 196 | 6.204933333 | metab_12605 | 223.1698711 | neg | HMDB0031550 | 5-Methyl-3-hexen-2-one | 0.8292 | 0.009224 |
| 197 | 6.2547 | metab_4895 | 347.3048685 | pos | HMDB0034146 | Octadecanamide | 1.2099 | 0.0001512 |
| 198 | 6.310466667 | metab_4842 | 258.242165 | pos | - | D-erythro-Sphingosine C-15 | 1.2473 | 0.005019 |
| 199 | 6.358466667 | metab_4778 | 550.3841851 | pos | - | PC(20:1/0:0) | 0.7521 | 0.003714 |
| 200 | 6.366466667 | metab_4770 | 470.3258282 | pos | HMDB0242385 | Cholylproline | 0.8038 | 0.00007147 |
| 201 | 6.383633333 | metab_11025 | 567.2625246 | neg | HMDB0040844 | (S)-Nerolidol 3-O-[a-L-rhamnopyranosyl-(1->2)-b-D-glucopyranoside] | 0.7354 | 0.000005404 |
| 202 | 6.391416667 | metab_12391 | 490.3536826 | neg | HMDB0242423 | Deoxycholylvaline | 0.7728 | 0.00007592 |
| 203 | 6.4144 | metab_12380 | 253.1805836 | neg | HMDB0035862 | (E)-6,10-Dimethyl-9-methylene-5-undecen-2-one | 1.5594 | 6.457E-07 |
| 204 | 6.430266667 | metab_4701 | 336.270963 | pos | HMDB0251559 | Docosahexaenoylethanolamine | 0.8152 | 0.002502 |
| 205 | 6.462033333 | metab_3357 | 470.3826879 | pos | HMDB0000513 | 5b-Cholestane-3a,7a,12a,23R,25-pentol | 0.8062 | 0.002806 |
| 206 | 6.4763 | metab_11077 | 570.3046426 | neg | HMDB0304815 | Val-Tyr-Leu-Arg | 1.4329 | 3.005E-07 |
| 207 | 6.530983333 | metab_12276 | 188.0168163 | neg | HMDB0252392 | Fluoroquinolone carboxylic | 0.8054 | 0.02949 |
| 208 | 6.538816667 | metab_12264 | 366.264279 | neg | HMDB0253296 | Hydroxytetradecenoylcarnitine | 0.7943 | 0.0002181 |
| 209 | 6.573516667 | metab_4558 | 229.1944764 | pos | HMDB0061780 | 1,1,2-Trimethyl-3,5-bis(1-methylethenyl)cyclohexane | 0.8006 | 0.000001258 |
| 210 | 6.577916667 | metab_11166 | 389.2309184 | neg | HMDB0249644 | Carboprost | 0.7209 | 0.000003907 |
| 211 | 6.581366667 | metab_4542 | 607.4532916 | pos | HMDB0294736 | DG(20:5(5Z,8Z,11Z,14Z,16E)-OH(18R)/0:0/12:0) | 1.2173 | 0.00005693 |
| 212 | 6.597316667 | metab_4536 | 767.5786353 | pos | HMDB0257015 | Quadazocine | 0.7139 | 0.00002104 |
| 213 | 6.609283333 | metab_12198 | 255.2325987 | neg | HMDB0034153 | Ethyl Myristate | 1.2154 | 0.004054 |
| 214 | 6.613183333 | metab_4522 | 588.3285924 | pos | HMDB0253209 | Homosalate | 0.7879 | 3.82E-08 |
| 215 | 6.679433333 | metab_12156 | 382.2958688 | neg | HMDB0248445 | Anisperimus | 0.7514 | 0.000001309 |
| 216 | 6.679433333 | metab_12157 | 353.2304083 | neg | LMFA03010044 | PGH1 | 0.7955 | 0.03657 |
| 217 | 6.684466667 | metab_4452 | 621.4686533 | pos | HMDB0034739 | Bullatanocin | 1.2745 | 0.03428 |
| 218 | 6.687216667 | metab_11232 | 521.3323987 | neg | HMDB0001959 | Gamma-Aminobutyryllysine | 1.2846 | 5.023E-07 |
| 219 | 6.687216667 | metab_15856 | 537.3229188 | neg | HMDB0015570;HMDB0001933 | Fusidic Acid | 1.2106 | 0.000004662 |
| 220 | 6.702783333 | metab_11237 | 351.2204969 | neg | HMDB0002776 | 13,14-Dihydro-15-keto-PGE2 | 1.4343 | 0.004951 |
| 221 | 6.710483333 | metab_11248 | 553.3533481 | neg | HMDB0010344 | Vitamin D2 3-glucuronide | 0.7066 | 1.251E-10 |
| 222 | 6.71605 | metab_4425 | 544.3392558 | pos | HMDB0010395 | LysoPC(20:4(5Z,8Z,11Z,14Z)/0:0) | 0.8308 | 0.007144 |
| 223 | 6.718266667 | metab_11253 | 439.2462641 | neg | HMDB0011154 | LysoPA(P-16:0/0:0) | 2.2869 | 0.0003433 |
| 224 | 6.723883333 | metab_1329 | 593.3436991 | pos | HMDB0242751 | Carbenoxolone | 0.4689 | 0.000006448 |
| 225 | 6.725966667 | metab_11263 | 418.332079 | neg | HMDB0241945 | N-Stearoyl Leucine | 0.7697 | 1.663E-07 |
| 226 | 6.725966667 | metab_12097 | 491.277488 | neg | HMDB0253026 | Phenylalanine amide | 1.3641 | 9.734E-08 |
| 227 | 6.747616667 | metab_4392 | 441.318652 | pos | HMDB0242065 | N-Eicosapentaenoyl Arginine | 1.443 | 4.825E-07 |
| 228 | 6.747616667 | metab_4389 | 647.484247 | pos | HMDB0035389 | Asitrilobin D | 1.3939 | 0.02223 |
| 229 | 6.77255 | metab_12067 | 655.3457865 | neg | HMDB0014736 | Labetalol | 1.4919 | 1.208E-11 |
| 230 | 6.786833333 | metab_3504 | 291.2439901 | pos | HMDB0031270 | 1-Nonene | 0.7878 | 0.0007108 |
| 231 | 6.803433333 | metab_11307 | 431.1889833 | neg | HMDB0042013;HMDB0247789 | Sofalcone | 0.77 | 0.00001532 |
| 232 | 6.810666667 | metab_3515 | 626.3651643 | pos | HMDB0034930 | (3beta,6beta)-Furanoeremophilane-3,6-diol 6-acetate | 1.2969 | 0.00000377 |
| 233 | 6.8262 | metab_3523 | 648.347052 | pos | HMDB0039275 | [6]-Gingerdione | 1.3151 | 0.000004873 |
| 234 | 6.850033333 | metab_12043 | 420.347846 | neg | HMDB0032001 | N-(2,4-Eicosadienoyl)piperidine | 0.6847 | 1.693E-08 |
| 235 | 6.85785 | metab_11325 | 431.2559379 | neg | HMDB0260512 | MG(20:3(6,8,11)-OH(5)/0:0/0:0) | 1.4245 | 9.245E-07 |
| 236 | 6.904983333 | metab_4300 | 677.5311879 | pos | HMDB0299664 | DG(i-18:0/18:1(12Z)-2OH(9,10)/0:0) | 1.3755 | 0.01096 |
| 237 | 6.943166667 | metab_11995 | 433.2044671 | neg | HMDB0061126 | 4,5-Dihydro-drospirenone-3-sulfate | 0.6923 | 0.000001925 |
| 238 | 7.029283333 | metab_11372 | 379.32112 | neg | HMDB0302983 | Tetracosanedioic acid | 0.7497 | 0.0001775 |
| 239 | 7.07585 | metab_11977 | 776.5133187 | neg | HMDB0260939 | PE(18:1(12Z)-O(9S,10R)/16:0) | 0.7357 | 0.000009587 |
| 240 | 7.091433333 | metab_11381 | 433.2715314 | neg | HMDB0242459 | (2-Acetyloxy-3-hydroxypropyl) (E)-octadec-9-enoate | 1.2756 | 0.001435 |
| 241 | 7.287216667 | metab_11945 | 355.3213166 | neg | HMDB0304091;HMDB0302963 | 22-Hydroxydocosanoic acid | 0.7986 | 0.00003809 |
| 242 | 7.341933333 | metab_11935 | 423.3476131 | neg | HMDB0031020 | 2-Dodecenal | 0.7725 | 0.00007659 |
| 243 | 7.9758 | metab_3825 | 337.2515159 | pos | HMDB0013627 | Cervonoyl ethanolamide | 0.8291 | 0.001001 |

**Table. S2.** The significant different metabolites between GCL and PH group. Variable importance in the projection (VIP) was obtained from OPLS-DA with a threshold of 1.0. P value was calculated from student's t-test. Fold change(FC) was calculated from the arithmetic mean values between GCL and PH group with a thresold of 1.2 or 0.83.

| **No.** | **Rt** | **Metabolite ID** | **m/z** | **Detection mode** | **HMDB_ID** | **Identification** | **FC** | ***P value*** |
| --- | --- | --- | --- | --- | --- | --- | --- | --- |
| 1 | 0.586 | metab_8353 | 112.087 | pos | HMDB0000870 | Histamine | 0.809 | 0.009 |
| 2 | 0.596 | metab_8970 | 249.050 | neg | HMDB0245528 | 1-((2S,4S,5R)-4-Hydroxy-5-(hydroxymethyl)tetrahydrofuran-2-yl)pyrimidine-2,4(1H,3H)-dione | 0.793 | 0.010 |
| 3 | 0.620 | metab_8996 | 202.119 | neg | HMDB0028846 | Glycyl-Lysine | 0.797 | 0.001 |
| 4 | 0.673 | metab_1545 | 232.140 | pos | HMDB0034326 | L-erythro-4-Hydroxyarginine | 0.794 | 0.002 |
| 5 | 0.705 | metab_14993 | 189.087 | neg | HMDB0031412 | 2-Amino-4-[(2-hydroxy-1-oxopropyl)amino]butanoic acid | 0.814 | 0.000 |
| 6 | 0.728 | metab_14949 | 246.109 | neg | HMDB0029075 | Threoninyl-Gamma-glutamate | 0.805 | 0.001 |
| 7 | 0.986 | metab_15860 | 269.088 | neg | HMDB0000197;HMDB0000190;HMDB0001311 | Lactic Acid | 0.785 | 0.001 |
| 8 | 0.986 | metab_14726 | 270.157 | neg | HMDB0028717 | Arginylproline | 0.749 | 0.002 |
| 9 | 1.048 | metab_9238 | 272.173 | neg | HMDB0028722 | Arginylvaline | 0.724 | 0.001 |
| 10 | 1.049 | metab_1709 | 301.107 | pos | HMDB0031897 | Phaseolic acid | 0.821 | 0.016 |
| 11 | 1.344 | metab_9354 | 216.099 | neg | HMDB0000856 | N-alpha-Acetyl-L-citrulline | 0.740 | 0.000 |
| 12 | 1.422 | metab_9365 | 267.146 | neg | HMDB0028889;HMDB0253179 | Histidinyl-Leucine | 0.752 | 0.001 |
| 13 | 1.647 | metab_9404 | 258.146 | neg | HMDB0028868 | Hydroxyprolyl-Lysine | 0.814 | 0.002 |
| 14 | 1.749 | metab_14454 | 205.065 | neg | HMDB0028973 | Methionyl-Glycine | 0.647 | 0.003 |
| 15 | 1.763 | metab_7400 | 207.079 | pos | HMDB0028847 | Glycyl-Methionine | 0.794 | 0.001 |
| 16 | 1.779 | metab_1915 | 93.070 | pos | HMDB0250648 | Cyclohexa-2,4-dienylmethanol | 1.220 | 0.049 |
| 17 | 1.804 | metab_9459 | 308.135 | neg | HMDB0028857 | Hydroxyprolyl-Arginine | 0.374 | 0.000 |
| 18 | 1.866 | metab_7358 | 306.165 | pos | - | Ser Ile Ser | 0.813 | 0.006 |
| 19 | 1.906 | metab_14411 | 575.149 | neg | HMDB0035872 | Oleuropein | 0.797 | 0.025 |
| 20 | 2.025 | metab_14261 | 429.126 | neg | HMDB0258126 | 6,8-Bis(sulfanyl)octanal | 1.295 | 0.000 |
| 21 | 2.123 | metab_7139 | 251.150 | pos | HMDB0028888 | Histidylisoleucine | 0.631 | 0.006 |
| 22 | 2.147 | metab_8731 | 259.092 | pos | - | Spongothymidine | 1.208 | 0.007 |
| 23 | 2.226 | metab_7120 | 331.197 | pos | - | Ala Leu Gln | 0.752 | 0.007 |
| 24 | 2.290 | metab_9587 | 292.166 | neg | HMDB0304786;HMDB0028958 | Lysylphenylalanine | 0.715 | 0.001 |
| 25 | 2.333 | metab_9593 | 329.183 | neg | HMDB0254713;HMDB0005764 | Melanostatin | 0.728 | 0.003 |
| 26 | 2.346 | metab_9596 | 243.146 | neg | HMDB0252056 | Ethyl N2-acetyl-L-argininate | 0.827 | 0.001 |
| 27 | 2.451 | metab_7039 | 168.029 | pos | HMDB0000232 | Quinolinic Acid | 0.779 | 0.001 |
| 28 | 2.451 | metab_7037 | 335.123 | pos | HMDB0246178 | 4-Aminophenylmannoside | 0.679 | 0.000 |
| 29 | 2.538 | metab_2120 | 307.064 | pos | HMDB0252787 | Glucose pyruvate acetate | 1.275 | 0.004 |
| 30 | 2.702 | metab_9628 | 265.119 | neg | HMDB0029005 | Phenylalanylthreonine | 0.788 | 0.002 |
| 31 | 2.732 | metab_9634 | 279.098 | neg | HMDB0011167 | L-beta-aspartyl-L-phenylalanine | 0.737 | 0.001 |
| 32 | 2.764 | metab_9642 | 461.127 | neg | HMDB0248814 | Azobilirubin | 0.769 | 0.006 |
| 33 | 2.961 | metab_14128 | 346.064 | neg | HMDB0254502 | Metamizol | 1.238 | 0.011 |
| 34 | 3.041 | metab_9681 | 157.134 | neg | HMDB0255016 | N-Acetyl-1,6-diaminohexane | 0.655 | 0.003 |
| 35 | 3.057 | metab_2364 | 378.117 | pos | HMDB0032057 | (E)-3-(2,3-Dihydroxyphenyl)-2-propenoic acid | 0.729 | 0.000 |
| 36 | 3.148 | metab_14060 | 247.112 | neg | HMDB0259742;HMDB0029133 | Valylmethionine | 0.806 | 0.000 |
| 37 | 3.160 | metab_6419 | 466.160 | pos | HMDB0253989 | Latrunculin a | 0.802 | 0.001 |
| 38 | 3.184 | metab_2406 | 139.123 | pos | HMDB0060677 | 2,6-Dimethylaniline | 1.302 | 0.025 |
| 39 | 3.270 | metab_474 | 377.181 | pos | HMDB0257277 | {1-[2-(4-Carbamimidoyl-benzoylamino)-propionyl]-piperidin-4-yloxy}-acetic acid | 0.827 | 0.004 |
| 40 | 3.421 | metab_6235 | 250.180 | pos | HMDB0036881 | Germacrone-13-al | 1.215 | 0.000 |
| 41 | 3.427 | metab_13921 | 388.125 | neg | HMDB0031999 | Casuarine 6-alpha-D-glucoside | 0.804 | 0.029 |
| 42 | 3.515 | metab_13874 | 230.996 | neg | HMDB0304906 | 4-Hydroxyphenylacetic acid sulfate | 1.224 | 0.011 |
| 43 | 3.526 | metab_6166 | 311.170 | pos | HMDB0029066 | Threonyllysine | 0.242 | 0.000 |
| 44 | 3.553 | metab_13853 | 377.183 | neg | HMDB0038980 | (4R,6S)-p-Menth-1-ene-4,6-diol 4-glucoside | 0.779 | 0.003 |
| 45 | 3.655 | metab_13792 | 340.059 | neg | HMDB0013854 | N4-Acetylsulfamethoxazole | 1.470 | 0.034 |
| 46 | 3.655 | metab_9946 | 320.161 | neg | HMDB0246810 | 5-Hydroxyprimaquine | 0.826 | 0.003 |
| 47 | 3.683 | metab_13778 | 290.089 | neg | HMDB0247293 | 7,8-Dihydro-2'-deoxyguanosine | 1.298 | 0.007 |
| 48 | 3.702 | metab_9963 | 275.125 | neg | HMDB0012880 | Acetamidopropanal | 0.235 | 0.007 |
| 49 | 3.703 | metab_8754 | 499.287 | pos | HMDB0002829;HMDB0004484 | Etiocholanolone glucuronide | 0.808 | 0.003 |
| 50 | 3.751 | metab_6041 | 403.269 | pos | HMDB0003252 | Thromboxane B2 | 1.215 | 0.038 |
| 51 | 3.771 | metab_10003 | 325.067 | neg | HMDB0001067;HMDB0255414 | N-Acetyl-1-aspartylglutamic acid | 0.654 | 0.012 |
| 52 | 3.872 | metab_2639 | 227.058 | pos | HMDB0242197 | (-)-2-Difluoromethylornithine | 1.214 | 0.020 |
| 53 | 3.897 | metab_13651 | 445.230 | neg | HMDB0256718 | Metapro | 0.776 | 0.001 |
| 54 | 3.957 | metab_13628 | 94.012 | neg | HMDB0038460 | Thiiranepropanenitrile | 0.812 | 0.005 |
| 55 | 3.986 | metab_13611 | 415.219 | neg | HMDB0251243 | Diethylaminoethyl-Sephacel | 0.780 | 0.002 |
| 56 | 4.023 | metab_10101 | 334.177 | neg | HMDB0243517 | Dimethylphysostigmine | 0.737 | 0.002 |
| 57 | 4.030 | metab_10104 | 220.065 | neg | HMDB0258091 | (2R)-2-Acetamido-5-hydroxy-2-(sulfanylmethyl)pentanoic acid | 0.822 | 0.001 |
| 58 | 4.030 | metab_13591 | 346.180 | neg | HMDB0011627 | Farnesylcysteine | 0.615 | 0.005 |
| 59 | 4.038 | metab_13588 | 362.172 | neg | HMDB0254077 | Libenzapril | 0.809 | 0.047 |
| 60 | 4.057 | metab_5903 | 394.233 | pos | HMDB0000374 | 17-Hydroxyprogesterone | 0.823 | 0.005 |
| 61 | 4.058 | metab_13577 | 441.199 | neg | HMDB0258096 | Adenosine, 8-(butylamino)-N-cyclopentyl- | 0.709 | 0.004 |
| 62 | 4.096 | metab_10131 | 401.204 | neg | HMDB0260384 | 9-(3-Imidazol-1-yl-2,6,6-trimethylcyclohexen-1-yl)-3,7-dimethylnona-2,4,6,8-tetraenoic acid | 0.771 | 0.003 |
| 63 | 4.106 | metab_5888 | 461.259 | pos | HMDB0242565 | Geneticin | 0.824 | 0.038 |
| 64 | 4.162 | metab_10154 | 394.161 | neg | HMDB0304025 | 16, 17-dihydro-16alpha, 17-dihydroxy GA9 | 0.790 | 0.013 |
| 65 | 4.202 | metab_2730 | 311.103 | pos | HMDB0060832 | Meta-O-Dealkylated flecainide lactam | 0.742 | 0.000 |
| 66 | 4.226 | metab_5841 | 315.195 | pos | HMDB0003034 | Prostaglandin D3 | 1.353 | 0.003 |
| 67 | 4.306 | metab_10200 | 305.070 | neg | HMDB0251458 | Diphenylhydantoic acid | 1.261 | 0.009 |
| 68 | 4.364 | metab_13458 | 314.114 | neg | HMDB0028830 | Glutamyltryptophan | 0.536 | 0.010 |
| 69 | 4.379 | metab_10220 | 389.171 | neg | HMDB0001220 | Prostaglandin E2 | 0.779 | 0.002 |
| 70 | 4.423 | metab_10230 | 360.194 | neg | HMDB0252196 | Fendiline | 0.724 | 0.003 |
| 71 | 4.496 | metab_10247 | 395.207 | neg | HMDB0247474;HMDB0002664 | Prostaglandin E3 | 1.227 | 0.001 |
| 72 | 4.548 | metab_10261 | 356.119 | neg | HMDB0061011 | N-desmethylalmotriptan | 0.559 | 0.005 |
| 73 | 4.556 | metab_2806 | 423.157 | pos | HMDB0030071 | Albafuran A | 0.681 | 0.027 |
| 74 | 4.564 | metab_10266 | 381.228 | neg | HMDB0039171 | 9alpha-(3-Methylbutanoyloxy)-4S-hydroxy-10(14)-oplopen-3-one | 1.253 | 0.001 |
| 75 | 4.584 | metab_13387 | 330.167 | neg | - | N-Acetylleucylalanylserine | 0.824 | 0.003 |
| 76 | 4.584 | metab_10270 | 289.140 | neg | HMDB0252495;HMDB0242138;HMDB0034879 | Fructosyl-lysine | 0.484 | 0.003 |
| 77 | 4.597 | metab_10275 | 361.093 | neg | HMDB0302539 | Neobyakangelicol | 0.786 | 0.042 |
| 78 | 4.629 | metab_10282 | 307.085 | neg | HMDB0249574 | Camonagrel | 1.292 | 0.004 |
| 79 | 4.709 | metab_793 | 315.195 | pos | HMDB0062410 | 5,6-epoxy,18R-HEPE | 1.254 | 0.002 |
| 80 | 4.735 | metab_13344 | 179.071 | neg | HMDB0059601 | Indan-1-ol | 0.810 | 0.000 |
| 81 | 4.754 | metab_13336 | 259.155 | neg | HMDB0035028 | (x)-2-Heptanol glucoside | 0.670 | 0.005 |
| 82 | 4.762 | metab_10321 | 395.207 | neg | HMDB0062297 | 9S-hydroxy-11,15-dioxo-5Z,13E-prostadienoic acid | 1.224 | 0.000 |
| 83 | 4.769 | metab_13330 | 429.235 | neg | HMDB0255670 | Nivacortol | 0.765 | 0.004 |
| 84 | 4.775 | metab_10324 | 367.212 | neg | HMDB0012110 | 5(6)-Epoxy Prostaglandin E1 | 1.243 | 0.000 |
| 85 | 4.807 | metab_10333 | 253.083 | neg | HMDB0000684 | L-Kynurenine | 0.814 | 0.012 |
| 86 | 4.829 | metab_8759 | 331.200 | pos | HMDB0014568;HMDB0242263;HMDB0003573 | Hyoscyamine | 1.405 | 0.026 |
| 87 | 5.038 | metab_13264 | 426.177 | neg | HMDB0255595 | Nifekalant | 1.607 | 0.000 |
| 88 | 5.046 | metab_13262 | 263.150 | neg | HMDB0000665 | Leucinic Acid | 0.733 | 0.003 |
| 89 | 5.052 | metab_10388 | 271.136 | neg | HMDB0032223 | Diisopentyl thiomalate | 0.620 | 0.004 |
| 90 | 5.052 | metab_13257 | 285.132 | neg | HMDB0244804 | 1,4,7,10,13,16-Hexaoxacyclooctadecane | 0.755 | 0.005 |
| 91 | 5.052 | metab_13258 | 261.029 | neg | HMDB0014787 | Stavudine | 0.822 | 0.000 |
| 92 | 5.133 | metab_10403 | 268.082 | neg | HMDB0243842;HMDB0013068 | Salsolinol-1-carboxylic acid | 0.622 | 0.000 |
| 93 | 5.227 | metab_10432 | 238.083 | neg | HMDB0062376 | 4-(Nitrosoamino)-1-(3-pyridinyl)-1-butanone | 1.206 | 0.013 |
| 94 | 5.271 | metab_880 | 417.249 | pos | - | Ile Val Trp | 0.810 | 0.001 |
| 95 | 5.302 | metab_14102 | 147.044 | neg | HMDB0000779 | 3-Phenyllactic Acid | 0.819 | 0.000 |
| 96 | 5.591 | metab_10530 | 236.092 | neg | HMDB0062175 | N-Lactoylphenylalanine | 0.822 | 0.001 |
| 97 | 5.656 | metab_15866 | 230.082 | neg | HMDB0062723 | N-hydroxy-4-aminobiphenyl | 0.771 | 0.006 |
| 98 | 5.830 | metab_10592 | 301.176 | neg | HMDB0257190 | Arginylphenylalaninamide | 0.741 | 0.002 |
| 99 | 5.837 | metab_13014 | 426.189 | neg | HMDB0241288 | 5-Hydroxydodecanedioylcarnitine | 1.235 | 0.000 |
| 100 | 5.963 | metab_10687 | 549.271 | neg | HMDB0246702 | Phorbol-12,13-dibutyrate | 0.714 | 0.001 |
| 101 | 6.112 | metab_12695 | 278.140 | neg | HMDB0034684 | Thalictroidine | 0.786 | 0.001 |
| 102 | 6.120 | metab_10830 | 973.466 | neg | HMDB0278358 | PI(5-iso PGF2VI/22:6(4Z,7Z,10Z,13Z,16Z,19Z)) | 1.236 | 0.007 |
| 103 | 6.120 | metab_12684 | 909.504 | neg | HMDB0285619 | PC(LTE4/14:0) | 1.272 | 0.000 |
| 104 | 6.151 | metab_5000 | 639.283 | pos | HMDB0259343 | Tyr-ile-gly-ser-arg | 1.310 | 0.027 |
| 105 | 6.205 | metab_10879 | 192.945 | neg | HMDB0246393 | 4-Chlorobenzoic acid | 1.440 | 0.000 |
| 106 | 6.366 | metab_4770 | 470.326 | pos | HMDB0242385 | Cholylproline | 1.344 | 0.000 |
| 107 | 6.462 | metab_3357 | 470.383 | pos | HMDB0000513 | 5b-Cholestane-3a,7a,12a,23R,25-pentol | 1.301 | 0.010 |
| 108 | 6.578 | metab_11166 | 389.231 | neg | HMDB0249644 | Carboprost | 1.264 | 0.002 |
| 109 | 6.718 | metab_11253 | 439.246 | neg | HMDB0011154 | LysoPA(P-16:0/0:0) | 0.511 | 0.001 |
| 110 | 6.724 | metab_1329 | 593.344 | pos | HMDB0242751 | Carbenoxolone | 1.479 | 0.007 |
| 111 | 6.765 | metab_12072 | 591.463 | neg | HMDB0250290 | Cis-Linoleic acid | 1.270 | 0.002 |
| 112 | 6.773 | metab_12067 | 655.346 | neg | HMDB0014736 | Labetalol | 0.799 | 0.004 |
| 113 | 6.787 | metab_3504 | 291.244 | pos | HMDB0031270 | 1-Nonene | 1.295 | 0.000 |
| 114 | 6.937 | metab_3561 | 617.475 | pos | HMDB0304033 | 18-hydroxyoleate | 1.211 | 0.046 |
| 115 | 7.091 | metab_11381 | 433.272 | neg | HMDB0242459 | (2-Acetyloxy-3-hydroxypropyl) (E)-octadec-9-enoate | 0.817 | 0.001 |
| 116 | 7.744 | metab_216 | 399.217 | pos | HMDB0002294 | Resolvin D2 | 0.801 | 0.000 |
